# “A draft *Musa balbisiana* genome sequence for molecular genetics in polyploid, inter- and intra-specific *Musa* hybrids”

**DOI:** 10.1186/1471-2164-14-683

**Published:** 2013-10-05

**Authors:** Mark W Davey, Ranganath Gudimella, Jennifer Ann Harikrishna, Lee Wan Sin, Norzulaani Khalid, Johan Keulemans

**Affiliations:** 1 Laboratory of Fruit Breeding and Biotechnology, Division of Crop Biotechnics, Department of Biosystems, Katholieke Universiteit Leuven, Willem de Croylaan 42, box 2427B-3001, Heverlee, Leuven, Belgium; 2 Centre for Research in Biotechnology for Agriculture and Institute of Biological Sciences, Faculty of Science, University of Malaya, Kuala Lumpur 50603, Malaysia

**Keywords:** (a)biotic stress, Banana, Fe’i, Genetic diversity, microRNA (miRNA), Molecular breeding, *Musa acuminata*, *Musa balbisiana*, Pisang Klutuk Wulung, Plantain, Polyploidy, Wild banana

## Abstract

**Background:**

Modern banana cultivars are primarily interspecific triploid hybrids of two species, *Musa acuminata* and *Musa balbisiana*, which respectively contribute the A- and B-genomes. The *M. balbisiana* genome has been associated with improved vigour and tolerance to biotic and abiotic stresses and is thus a target for *Musa* breeding programs. However, while a reference *M. acuminata* genome has recently been released (Nature 488:213–217, 2012), little sequence data is available for the corresponding B-genome.

To address these problems we carried out Next Generation gDNA sequencing of the wild diploid *M*. *balbisiana* variety ‘Pisang Klutuk Wulung’ (PKW). Our strategy was to align PKW gDNA reads against the published A-genome and to extract the mapped consensus sequences for subsequent rounds of evaluation and gene annotation.

**Results:**

The resulting B-genome is 79% the size of the A-genome, and contains 36,638 predicted functional gene sequences which is nearly identical to the 36,542 of the A-genome. There is substantial sequence divergence from the A-genome at a frequency of 1 homozygous SNP per 23.1 bp, and a high degree of heterozygosity corresponding to one heterozygous SNP per 55.9 bp. Using expressed small RNA data, a similar number of microRNA sequences were predicted in both A- and B-genomes, but additional novel miRNAs were detected, including some that are unique to each genome. The usefulness of this B-genome sequence was evaluated by mapping RNA-seq data from a set of triploid AAA and AAB hybrids simultaneously to both genomes. Results for the plantains demonstrated the expected 2:1 distribution of reads across the A- and B-genomes, but for the AAA genomes, results show they contain regions of significant homology to the B-genome supporting proposals that there has been a history of interspecific recombination between homeologous A and B chromosomes in *Musa* hybrids.

**Conclusions:**

We have generated and annotated a draft reference *Musa* B-genome and demonstrate that this can be used for molecular genetic mapping of gene transcripts and small RNA expression data from several allopolyploid banana cultivars. This draft therefore represents a valuable resource to support the study of metabolism in inter- and intraspecific triploid *Musa* hybrids and to help direct breeding programs.

## Background

Bananas and plantains (Musaceae, Zingiberlaes, *Musa* ssp., ‘bananas’) are giant monocotoyledenous herbs, which originated in Southeast Asia and the western Pacific. They were one of the first crops to be domesticated, and are now widely distributed throughout the subtropics where they constitute a major staple food for millions of people [1]. Indeed, bananas are the World’s most important fruit and rank fourth in the list of global food crops [2], but with the important caveat that 80% of the production is for local consumption [3].

There are four genomes present within *Musa* spp., corresponding to the genetic constitutions of the four wild Eumusa species i.e. *Musa acuminata* (A-genome, 2n = 2x = 22), *Musa balbisiana* (B-genome, 2n = 2x = 22), *Musa schizocarpa* (S genome, 2n = 2x = 22) and the *Australimusa* species (T genome, 2n = 2x = 20). Within *M*. *acuminata*, a number of subspecies are also recognized which have been further classified into four subgroups on the basis of DNA markers i.e. banksii, zebrina, malaccensis and burmannica/burmannicoides [4-6]. Despite evidence of interspecific genetic variation, there has been no subspecies classification within *M*. *balbisiana*[7]. Members of the *Australimusa* sections have a basic chromosome number of 2n = 20, and comprise seven species one of which contains edible parthenocarpic types known as ‘Fe’i’ bananas. Members of this Fe’i group are notable for their upright fruit and in some cases exceptionally high fruit provitamin A carotenoid contents [8,9].

Cultivated bananas are parthenocarpic, generally seedless and vegetatively propagated hybrids that have arisen primarily as a result of hybridizations between wild diploid *M*. *acuminata* and *M*. *balbisiana* species [10-12]. Intraspecific hybridizations within *M. acuminata*, and interspecific hybridizations between *M. acuminata* and *M*. *balbisiana* have naturally resulted in various combinations of these A- and B -genomes which are classified into six groups (AA, AAA, AB, AAB, ABB and ABBB) [11]. The majority of edible cultivars though are allopolyploid triploids with a genome constitution of AAA (dessert banana), AAB (plantains) and ABB (cooking bananas).

It has also become increasingly clear that there is no simple division of parental A- and B- chromosomes during hybridization, and several research groups have provided evidence for pairing and recombination between homeologous A- and B- chromosomes [13,14]. Similar events have been observed in allopolyploids of other species [15]. It seems likely therefore that current interspecific triploids have arisen from one or more steps of (re)combination and exchange of chromosomal segments between the A- and B-genomes [13,16,17]. As a result, most if not all *Musa* cultivars probably have genomes consisting of different proportions of the A- and B-genome. A similar process of hybridization between subspecies of *M. acuminata* probably also underlines the evolution of the edible AA and AAA types. A consequence of these recombination events is that the hybrid genomes contain an unbalanced number of A- and B-genome alleles [18]. This clearly complicates genetic studies of trait inheritance, as well as the development and application of molecular marker technologies [17].

Currently, over 90% of commercial export dessert bananas are produced from a single cultivar, namely ‘Cavendish’ (AAA). Unsurprisingly, this dependency on a single cultivar and the consequent lack of genetic variation in production systems, has resulted in a crop which is potentially highly susceptible to disease pandemics. Pandemics are also not without precedent in banana, and in the 1950’s, ‘Panama Disease’ (‘Fusarium wilt’) caused by the soil-born fungus *Fusarium oxysporum* was responsible for entirely wiping out commercial banana production, which at that time was dependent on the cultivar ‘Gros Michel’ (AAA). By necessity, ‘Gros Michel’ was replaced by the Fusarium-resistant cultivar ‘Cavendish’ , but unfortunately, in the intervening years new strains of *F. oxysporum* have evolved, (*F. oxysporum* f. sp. cubense race 4, ‘Tropical race 4’), that have overcome this natural Cavendish resistance [19]. Currently, the other major disease threat is that posed by *Mycosphaerella fijiensis* (‘Black Sigatoka’). Chemical control of Fusarium wilt is ineffective, and even though ‘Black Sigatoka’ can be controlled by frequent (up to 50) fungicide applications per year, these are socio-economically and environmentally inappropriate, and require integrated strategies to avoid the development of fungicide resistance in the pathogen [20,21].

Consequently, there is currently much interest in developing sustainable solutions to these disease threats through the introgression of novel resistance loci and the development of new, disease resistant varieties. However the largely sterile nature of the majority of commercial varieties means that banana breeding is both lengthy and time-consuming. Therefore the application of molecular marker technologies for the identification of trait-marker associations, and high throughput genotyping technologies can greatly accelerate the whole breeding cycle through marker assisted selection (MAS) [22]. In this regard the recent publication of a high quality 523 Mb draft reference genome for the doubled haploid genome of the wild *M. acuminata* variety ‘Pahang’ (AA), represents a major advance in this field [14]. The variety ‘Pahang’ is member of the subspecies malaccensis that contributed one of the three *M. acuminata* genomes for ‘Cavendish’ [4]. This invaluable resource therefore provides for the first time a complete catalogue of all predicted genes, transcripts and markers in *Musa*, and can greatly facilitate and accelerate the search for novel genes, transcripts, allelic variants etc., for important biological processes. It also opens up the prospects for the rapid development of high-throughput molecular tools for *Musa* improvement. Despite this, there is also an urgent need for the development of similar resources for the *Musa* B-genome, to be able to identify and exploit *M. balbisiana* accessions with resistance to various abiotic and biotic stresses including osmotic and cold stresses [23-26] as well as for vigour [27,28].

The aim of this work therefore was to sequence and assemble a draft genome of the wild *M. balbisiana* diploid variety ‘Pisang Klutuk Wulung’ (‘PKW’ , B-genome) for use in comparative transcriptomics and genomics studies of interspecific triploid and tetraploid hybrids. PKW is one of the possible ancestral parents of the B-genome present in cultivated triploids, and has for example has been shown to have very strong partial resistance to black leaf streak virus (http://www.proMusa.org/tiki-index.php?page=Pisang+Klutuk+Wulung). Other *M. balbisiana* cultivars have demonstrated resistance to Xanthomonas [29], and are considered to be more drought tolerant [24,26]. The utility of the PKW B-genome sequence generated here was validated by examining the distribution of CDS, EST and RNA-read mappings from a range of genetically diverse interspecific triploid cultivars across the combined A- and B-genomes, by characterising the predicted miRNA sequences encoded in both A- and B-genomes and by prediction of miRNA targets in both genomes.

Improved insights into genome structure, allelic diversity and regulatory elements within *M. balbisiana* spp. will help in the design and application of breeding strategies for novel banana cultivars with improved stress resistance and quality traits.

## Methods

### Plant materials

Sterile plantlets of the wild diploid *M. balbisiana* variety ‘Pisang Klutuk Wulang’ (‘PKW’ , BB genome), were obtained from the International Transit Centre, KU Leuven (reference number ITC 1587), and healthy leaf material collected for gDNA isolation from 8-week old, greenhouse grown plants. RNA was extracted from lyophilised fruit pulp samples from the following cultivars; ‘Yangambi-km5’ (AAA), ‘Gros Michel’ (AAA), ‘Batard’ (AAB), ‘Mbouroukou-3’ (AAB), ‘Iholena Lele sub var 'Long Peduncular'’ (AAB), and ‘Karat’ (Fe’i, *Australimusa*).

Banana root tissues were prepared from clonal tissue-cultured plantlets of *M. acuminata* cultivar ‘Berangan’ (AAA), when 6-8 cm tall, with three fully-expanded leaves and healthy roots. Root tissues were pooled from 3–4 plantlets for RNA isolation and small RNA sequencing. Banana embryogenic suspension cell samples were prepared from embryogenic callus induced from immature male flowers of ‘Berangan’ as described by [30]. Newly initiated suspension cells were used for RNA isolation and small RNA sequencing.

### gDNA extraction

Genomic DNA (gDNA) was extracted from young leaves essentially as according to Michiels et al. [31].

### RNA extraction

Total nucleic acids were extracted from lyophilized fruit pulp samples, from 3 individual fruit per cultivar using the Tris-LiCl procedure of Tattersall et al. [32], modified as previously described [33]. Equivalent amounts of RNA from each sample were combined per cultivar. Total nucleic acids were isolated from banana root tissues and embryogenic suspension cells using a modified CTAB nucleic acid isolation method [34].

### RNA and DNA quality

Concentrations of purified nucleotides were determined at 260 nm using a NanoDrop 2000 Spectophotometer (Thermo Scientific) and purity assessed at an absorbance ratio of 260/280 nm and 260/230 nm. RNA integrity was confirmed by agarose gel electrophoresis and on an Agilent 2100 Bioanalyzer (Agilent Technologies, Inc.). Only samples with high RNA integrity number (RIN ≥ 8) were used for RNA sequencing. A total of 2 μg of purified gDNA and of each combined RNA/DNA sample was precipitated in ethanol and used for sequencing.

### RNA and DNA sequencing

Sequencing of both RNA and DNA samples was carried out at the Genomics Core sequencing facilities of the Katholieke Universiteit Leuven (http://www.uzleuven.be/genomicscore/), using an Illumina HiSeq 2000 II instrument. Small RNA libraries were sequenced using an Illumina HiSeq 2000 II at BGI, Shenzhen.

### Illumina paired-end cDNA library construction and sequencing

The cDNA libraries were constructed using the TruSeq™ RNA Sample Preparation Kit (Illumina, Inc.) according to the manufacturer's instructions. Poly-A containing mRNA was purified from 2 μg of total RNA using oligo(dT) magnetic beads and fragmented into 200–500 bp pieces using divalent cations at 94°C for 5 min. The cleaved RNA fragments were copied into first strand cDNA using SuperScript II reverse transcriptase (Life Technologies, Inc.) and random primers. After second strand cDNA synthesis, fragments were end repaired, a-tailed and indexed adapters were ligated. The products were purified and enriched with PCR to create the final cDNA library. The 6 tagged cDNA libraries were pooled in equal ratios and used for 2 × 100 bp paired-end sequencing on a single lane of the Illumina HiSeq2000 II.

### Sequence data processing

Sequencing data was provided as fastq files and unless otherwise mentioned, all data processing steps were carried out using the CLC Genomics Workbench software package v 6.01. Raw reads were uploaded to GenBank and are accessible via accession number SAMN02333823.

### Mapping PKW gDNA reads to the reference A-genome

The raw data was first trimmed to remove low quality bases and the trimmed PKW gDNA reads then aligned to the publically available *M. acuminata* ‘Pahang’ doubled haploid A-genome assemblies available from http://banana-genome.cirad.fr/download.php. This consists of 11 chromosomes together with one sequence containing concatenated unassembled contigs, each separated by 100 ‘N’s’. Reads were aligned using the settings; mismatch cost (2), insertion cost (3) deletion cost (3), length fraction (0.9) and similarity fraction (0.9). Reads mapping equally well to two positions were assigned randomly. Following mapping, the consensus sequences were extracted and served as the PKW consensus reference genome for further treatments. For chromosomes 1 – 11, during extraction of the consensus PKW genome sequence, regions of 0 read coverage (e.g. gaps etc.) are removed to produce a single continuous sequence. For the large unmapped chromosome, the consensus PKW sequence was extracted using ‘N’ ambiguity symbols to fill in gap regions, as otherwise unrelated genic sequences could be concatenated together allowing ‘bridging’ of reads across unrelated genic sequences.

### Mapping RNA *de novo* assembled transcripts, CDS and unigenes to gDNA contigs and genome sequences

RNA reads (de novo assembled transcripts, CDS, unigenes), were aligned to the PKW genome or gDNA contig data using the ‘large gap’ mapping function within the CLC Genomics Workbench, using the following settings; Maximum number of hits for a segment = 10, Maximum distance from seed = 50,000, Key Match _Mode = random, Mismatch cost = 2, Insertion cost = 3, Deletion cost = 3, Similarity = 0.8, Length fraction = 0.9. The large gap mapper function aligns reads to a reference sequence, while allowing for large gaps in the mapping. It is therefore able to map reads that span introns without requiring prior transcript annotations or for the detection of large deletions in genomic data. Additional details can be found http://www.clcbio.com/white-paper.

### B-genome annotation

*Ab initio* gene prediction was carried out using the FGENESH software, available online from http://linux1.softberry.com/all.htm and using the default parameters and the ‘monocot model plant’ parameters. The list of predicted PKW gene models was then blasted against the NCBI nr protein database and gene ontology terms assigned using the Blast2Go software (Conesa et al., [35]; Götz et al., [36]). Repeats were annotated by BLAST against the repetitive part of the *Musa* genome containing 1902 sequences which were retrieved from a published report [37]. Evaluation of the PKW B-genome gene model set took place by large-gap mapping of available CDS, and EST resources within CLC Genomics Workbench. These resources consisted of the ‘Pahang’ consensus CDS set, an in-house *Musa* unigene set of 22,205 sequences derived from the Syngenta *M. acuminata* 3’ EST database [33], transcript sets generated from the *de novo* assembly of Illumina 100 bp paired end RNA reads from 6 *Musa* cultivars.

### *De novo* assembly

All the trimmed, PKW gDNA reads were *de novo* assembled using the default settings in CLC Genomics Workbench with the settings as follows; Word size: 25, Bubble size: 50, Minimum contig length = 200, Mismatch cost = 2, Insertion cost = 3, Deletion cost = 3, Length fraction = 0.5, Similarity fraction = 0.8. In addition the ‘scaffolding’ option was used to take advantage of the paired read data, and reads were mapped back to the contigs generated to validate the sequences. The same parameters were also used for the *de novo* assembly of PKW gDNA reads that could not be mapped to the ‘Pahang’ reference genome, and for the *de novo* assembly of all RNA transcript data from the 5 triploid hybrids and the diploid Fe’i variety.

### Variant analysis

Sequence variant analysis was carried out on both the RNA- and DNA-mappings using the ‘Probabilistic Variant Detection’ plugin in CLC Genomics Workbench, with settings specifying a minimum read coverage of 10 and a variant probability of >90%. The maximum expected number of variants was set as 2 or 3 according to the ploidy level of the samples and variants were calculated using either all the mapped reads, or only using the uniquely mapped reads.

### Repeat annotation

This was performed on both the ‘Pahang’ A-genome and the PKW consensus B-genomes (our data) as well as *de novo* assembled contigs with Repeat Masker V4.0.3 software tool (http://www.repeatmasker.org), [38] using RMBLAST 2.2.27 as the engine and using the customized library of *M. acuminata* repeats (1903 sequences) from Hribova et al. 2010 [37]. SSR detection was carried out using Tandem Repeat Finder (TRF) software [39] and TRAP [40] using the default parameters.

### microRNA (miRNA) and target prediction

miRNA prediction was performed using miRDeep2 tool [41] using scripts modified according to the criteria set for plant genomes [42]. A non-redundant query set of small RNA reads was compiled from root and embryogenic cell suspension and included all 235 miRNA sequences reported for the *Musa* ‘Pahang’ doubled haploid A-genome retrieved from the banana genome database [14], and publically available small RNA data from *M. acuminata* ‘Calcutta 4’ leaf, flower and fruit tissues (sequenced within the framework of a NSF project http://smallrna.udel.edu). Small RNA reads were trimmed to remove adapter sequences and low quality reads. Reads were then mapped to the Rfam database [43] using Bowtie [44]. Matches with tRNA, rRNA, small nucleolar RNA and sequences below 19 or above 24 nucleotides in length were not considered for further analysis. The query miRNA data set was mapped separately to the A-genome [14] and the draft PKW B-genome developed here. Regions of 300 nt surrounding the matched position of each read were excised from the genome sequences, and then RNAfold software [45] used to predict sequences able to form stem-loop structures, using default options, whilst ‘Randfold’ [46] was used to calculate p-values for potential miRNA precursors predicted by the ‘miRDeep2’ algorithm. The candidate miRNA precursors selected had the following features; a predicted stem loop structure of 75 nt and a bulge-loop size of less than 6 nt; the mature miRNA was within the stem region of the precursor; less than four mismatches were allowed between the mature miRNA:miRNA* duplex; miRNA and miRNA* were on opposite arms of the precursor forming a duplex with 3’ overhangs; the predicted minimum folding energy (MFE) was between -15 kcal/mol to −47.2 kcal/mol. Known miRNA sequences/homologues were annotated by BLASTn comparison [47] to mature and stem loop miRNA sequences from miRBase v19 [48]. Predicted miRNA were considered novel if they had no match (allowing for a maximum of 2 mismatches i.e. n/n, (n-1)/n, (n-2)/n nucleotide matches, n = length of mature miRNA) to any entry in miRBase (release 19) and PMRD (accessed, February 2013). Novel *Musa* miRNA sequences not present in either miRBAse or PMRD databases, were arbitrarily named starting at ‘1’ and using the miRBase species based name format. For miRNA families observed to be present in both A- and B-genomes, paralogous miRNA loci counts in each *Musa* genome were estimated based on the 300 nt precursor regions predicted by miRDeep2. miRNA targets were predicted with ‘psRNAtarget’ online server (http://plantgrn.noble.org/psRNATarget/) [49] with default options.

## Results and discussion

The majority of edible cultivated banana varieties are inter- and intra-specific triploid hybrids between varieties of two wild diploid *M. acuminata* and *M. balbisiana* species. To be able to carry out molecular genetic studies in these cultivars therefore it is necessary to have a reference B-genome. For these reasons we carried out Illumina 100 bp paired end gDNA sequencing of the wild diploid *M*. *balbisiana* variety ‘Pisang Klutuk Wulang’ (PKW), which is considered by many in the *Musa* research community to be one of the possible ancestors of the modern AAB hybrids.

### PKW gDNA Read Mapping to the A-genome

Over 281 M trimmed Illumina gDNA reads were mapped against the 12 reference A-genome chromosomal sequences. An overview of the quality parameters of these reads is supplied in Additional file 1. The results following alignment show that 86.9% of all the PKW gDNA reads successfully mapped to the A-genome, with a mean read coverage per reference A chromosome, of 41.4x (Table 1). The resulting consensus PKW genome sequences derived from these mappings were on average 78.9% of the length of the A-genome, with the greatest difference being found for chromosome 10 which was only 74.9% of the size of the corresponding ‘Pahang’ chromosome 10 (Table 1). The results from chromosome 12 (B-chrUn_random) were not included in this calculation because this ‘chromosome’ consists of non-assembled contigs, concatenated together with spacer ‘N’s’. Therefore, while the assembled PKW consensus sequence for ‘B-chrUn_random’ derived from read mapping is 80.01 Mb, for molecular genetic studies, all gaps were filled with ‘N’s to prevent artificial concatenation and ‘bridging’ of mapped reads across unrelated genic regions, giving a working sequence length of 141.13 Mbp, and a working PKW consensus genome size of 402.5 Mbp.

**Table 1:** overview of mean mapped read depth per chromosome, and the derived B-genome chromosome lengths following alignment to the A-genome

**Sequence**	**‘Pahang’ Reference length/bp**	**Mapped reads count**	**Mean mapped read depth**	**‘PKW’ mapped read length/bp**	**% of A -genome coverage**
chr1	27,573,629	11,164,973	36.9	22,038,404	79.9
chr2	22,054,697	8,996,066	37.1	17,349,238	78.7
chr3	30,470,407	12,275,431	36.6	24,161,952	79.3
chr4	30,051,516	13,632,301	41.3	24,656,528	82.0
chr5	29,377,369	12,398,532	38.2	23,648,591	80.5
chr6	34,899,179	16,499,244	43.0	27,831,592	79.7
chr7	28,617,404	13,123,105	42.1	22,212,853	77.6
chr8	35,439,739	14,663,790	37.7	27,665,716	78.1
chr9	34,148,863	14,480,461	38.5	25,900,723	75.8
chr10	33,665,772	14,673,154	39.6	25,230,959	74.9
chr11	25,514,024	11,897,139	42.3	20,721,546	81.2
chrUn_random	141,147,818	100,613,680	64.2	80,013,141*	56.7*
**total counts / averages**	**472,960,417**	**244,417,876**	**41.4**	341,431,243*	78.9**

*The assembled PKW consensus sequence for ‘B-chrUn_random’ derived from read mapping is 80.01 Mb, but for molecular genetic studies, all gaps were filled with ‘N’s to prevent artificial concatenation and ‘bridging’ of mapped reads across unrelated genic regions. Therefore the working sequence length is 141.13 Mbp, essentially the same as for the homoeologous A-genome sequence, and the working PKW consensus genome length is 402.547 Mbp.

**The mean% of A-genome chromosome length is calculated only for chromosomes 1 – 11.

This difference in size between the A- and B-genomes was largely expected as flow cytometric analyses of five *M. acuminata* genotypes (including DH ‘Pahang’) and representing 4 different subspecies, and of four *M. balbisiana* genotypes (including PKW) indicate that the haploid B-genome size is on average only 90% of the size of the A-genome [50-52]. However according to recent work from Cizkova et al. [50] the PKW haploid genome is actually 93.3% of the size of DH ‘Pahang’ with a sizes of 554 and 594 Mb respectively. This value for DH Pahang is slightly higher than the 523 Mb reported by D’Hont et al. 2012 [14]. The sequenced ‘Pahang’ genome size of 473 Mb, represents an assembly of ~90% of the total *M. acuminata* genome, of which only 323 Mb is anchored to the 11 chromosomes [14]. If the expected PKW *M. balbisiana* genome size is 93.3% of ‘Pahang’ then we would expect to generate a consensus length of in the region of 0.93 x 473 or 440 Mb. By analogy therefore, our consensus PKW B-genome size of 341.4 Mb represents ~78% of the expected PKW B-genome size.

### *De novo* assembly of unmapped gDNA reads

A total of 36.8 M gDNA reads (36.3 Gbp), remained unmapped after alignment to the A-genome. These could represent reads from regions that are structurally highly divergent from the A-genome so to test for the presence of unique, genic B-genome regions, the unmapped reads were *de novo* assembled into 63,245 contigs (Additional file 2: Table S2), and the presence of genic sequences tested for by large gap mapping of *Musa* unigene and reference CDS sequences, followed by a round of transcript detection. In total, 58,746 reads were used, but only 28 sequences actually mapped to these contigs. We can therefore conclude that the unmapped gDNA reads do not contain any significant gene rich regions, and that essentially all genic regions are retained in the consensus PKW B-genome sequence. An overview of the repeats annotation of these contig sequences is provided in Additional file 3: Table S3.

### *De novo* assembly of gDNA reads

We also carried out *de novo* assembly of all gDNA reads, independent of a reference sequence. Here, over 96% of the 281 M trimmed reads, representing 27.4 Gbp of nucleotide sequence were assembled into 180,175 contigs with a total length of 339.3 Mb, an N50 of 7,884 bp, and an average contig length of 1,883 bp (Additional file 4: Table S4). The accumulated assembled contig length of 339.3 Mb is very similar to the consensus read mapping length of 341 Mb, but due to its much more fragmented nature (78.2% of these contigs were less than 1 kb in length) this resource is much more difficult to utilize. To evaluate the set of PKW gDNA contig sequences, the *Musa* reference CDS set was mapped to the PKW contig set as well to as the consensus PKW B-genome. In the case of the consensus PKW B-genome 32,192 (88% of total) *Musa* CDS were successively mapped, corresponding to 25,565 individual transcripts. In the case of the gDNA contig set, 71% of the CDS could be mapped (25,694 CDS), and a total of 21,272 individual transcripts were identified (data not shown). These data indicate therefore that simply mapping the gDNA reads to the A-genome and extracting the consensus sequence is the most effective way to generate a draft working *M. balbisiana* genome.

### Evaluation/characterisation of the PKW B-genome assembly

**Figure 1:**
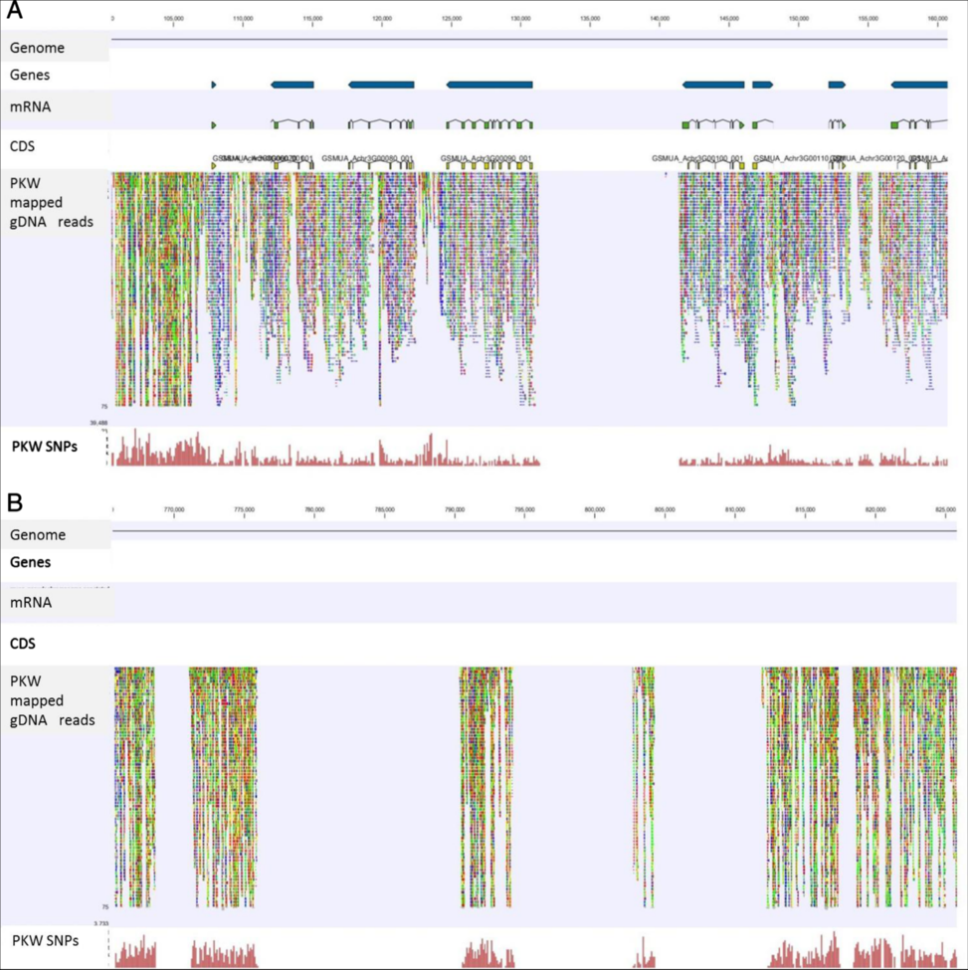
**Visualisation of ‘Pisang Klutuk Wulung’ (PKW) gDNA reads mapped to the reference ‘Pahang’ A-genome. (A)** Visualization of PKW gDNA read mappings to the reference ‘Pahang’ A-genome. Individual annotation tracks representing the reference genome, predicted genes, mRNA, CDS, read mapping and SNPs are indicated. Blue coloured reads represent unbroken 100 bp read pairs, single ‘forward’ reads are red, and ‘reverse’ reads in green. Non-specifically-mapped reads are coloured orange. **(B) **Visualization of PKW gDNA read mappings to an intergenic region of the reference ‘Pahang’ A-genome. Blue coloured reads represent unbroken 100 bp read pairs, single ‘forward’ reads are red, and ‘reverse’ reads in green. Non-specifically-mapped reads are coloured orange.

A visual inspection of the gDNA mappings to the reference A-genome clearly demonstrates that there are many regions of structural variance between the two genomes (Figure 1). However in general, the gene-rich regions seem to be well-conserved, as evidenced by the higher percentage of unbroken paired reads (in blue) in these regions. For example, direct transfer of annotations from the A-genome to the new PKW B-genome results in the transfer of 36,483 gene sequences (99.8%), indicating that regions homologous to essentially all genic regions of the A-genome are present in the PKW B-genome. Intergenic/non-transcribed regions by comparison typically contain a much higher proportion of unpaired, broken reads and more sequence variants.

While these results demonstrate that there are large regions with a high degree of homology across the *M. acuminata* and *M*. *balbisiana* genomes, it is important to realize that with this read mapping approach we cannot determine whether large scale genomic rearrangements such as insertions, inversions, transversions etc. have taken place. Previous work in banana however, suggests that gene order is likely to have been preserved - at least over short regions. For example, a comparison of 1.4 Mb of orthologous BAC clones from *M. acuminata* (cv. ‘Madang’), and *M*. *balbisiana* (cv. ‘Pisang Klutuk Wulung’), showed a high degree of microsynteny with preservation of gene order, and 96 – 96.3% sequence identity within genic regions [53,54]. The same authors also reported that predicted gene structure was good for well-conserved homologous genes, but that discrepancies were detected in the gene predictions of those orthologous BACs whose protein products had no match in public databases (i.e. hypothetical protein genes).

### Variant detection in the PKW B-genome

In total, 20,657,389 sequence variants were detected in the PKW B-genome relative to the reference doubled haploid ‘Pahang’ A-genome based on only the uniquely mapped reads (Additional file 5: Table S5). Of these 18,868,899 were single nucleotide variants (SNVs or SNPs), 815,805 insertions and 972,588 deletions. From the list of SNVs 8,738,760 were homozygous variants which therefore represent sequence differences from ‘Pahang’. The remaining 10,130,236 were heterozygous variants and therefore represent allelic variation and the degree of heterozygosity present in the PKW B-genome. On the basis of the total consensus PKW genome size of 341,431,243 bp, this heterozygosity corresponds to a SNP frequency of 1 variant every 33.7 bp, or 2.97%. The number and densities of sequence variants present in heterozygotic eukaryotic genomes varies enormously according to the species, whether they are obligate out-crossers or not, the number and genetic diversity of the cultivars assessed, and whether coding or noncoding regions are being considered. For example, in maize *Zea mays* L.) the SNV density was 1 SNV per 124 bp of coding sequence, and 1 per 31 bp in non-coding regions [55]. Visual inspection of Figure 1 also confirms that SNV density is higher in the intronic regions of the PKW B-genome than in the exons, and that there is also a higher SNV frequency in the non-transcribed regions. Similar conclusions were reached by Boonruangrod et al. in their comparison of the rDNA sequences of *M. acuminata* type ‘Calcutta4’ and ‘Yangambi KM5’ with the wild type *M*. *balbisiana* accession ‘Tani [5].

By comparison, our data indicate that the PKW gDNA sequence differs from the A-genome at a frequency of 1 (homozygous) SNV every 39.1 bp (2.6%). This is much higher than estimates for interspecies SNP variation in rice, where a comparison of the *O. indica* and *O. japonica* genome sequence data found a SNP frequency of around 0.4%, [56]. However differences between *M. acuminata* and *M. balbisiana* are comparable to frequencies measured in Eucalyptus species (between one SNP per 16 bp to one per 33 bp were reported [57]), *Quercus crispula* (one SNP in 25 bp [58]) and *Populus tremula* (one SNP per 60 bp [59]). Estimates for the degree of interspecific variation in *Musa* are likely to grow as more cultivars and species are sequenced.

### Repeat detection and annotation

In total, repetitive regions were found to occupy 26.85% (108.1 Mbp) of the PKW consensus B-genome, which is similar to the 27.76% reported by D’Hont et al. for the A-genome [1]. Annotation of the repetitive sequences of the B-genome showed that overall, the numbers of repeat elements is slightly higher in the B-genome and that the *Ty1/copia* and *Ty3/Gypsy* repeats dominate, representing 18.8% and 6.3% of the genome respectively (Table 2). Whilst the numbers of Non-LTR transposons (LINE), DNA transposons (clDNA and DNAhat) and Satellite repeats (Type 1 and Type 2) are similar in both A- and B-genomes and represent less than 1% of the total consensus B-genome sequence, the LTR transposons (*Ty1*/*copia* and *Ty3*/*Gypsy*) are more abundant in the B-genome.

**Table 2:** Overview and classification of the repeats present in the ‘Pahang’ (Musa A) and ‘Pisang Klutuk Wulung’ (PKW, Musa B) genomes

	**Musa A**			**Musa B**		
	**‘Pahang’**			**PKW**		
**Class**	**Count**	**Bp**	**%**	**Count**	**Bp**	**%**
Ty1/Copia	5,606	3,158,199	0.67%	5,616	2,760,972	0.69%
copia/Angela	3,2073	20,697,639	4.38%	32,056	19,380,064	4.81%
Copia/SIRE1Maximus	90,910	62,820,929	13.28%	97,868	49,333,251	12.26%
Copia/Tnt1	4,191	5,137,617	1.09%	4,377	4,320,053	1.07%
Ty3/Gypsy	6,236	6,717,506	1.42%	6,542	5,554,874	1.38%
Gypsy/CRM	1,051	1,124,528	0.24%	973	1,016,030	0.25%
Gypsy/Galadriel	1,992	2,997,110	0.63%	2,244	2,739,827	0.68%
Gypsy/Galadriel-lineage	1	28	0.00%	2	296	0.00%
Gypsy/Reina	16,445	11,955,226	2.53%	15,882	10,331,187	2.57%
Gypsy/Tekay	9,234	7,545,095	1.60%	9,245	5,851,644	1.45%
LINE	2,868	1,824,495	0.39%	2,544	1,580,226	0.39%
RE	14,494	5,415,085	1.14%	13,794	3,525,005	0.88%
Satellite/Type1	274	523,572	0.11%	299	484,199	0.12%
Satellite/Type2	68	103,955	0.02%	41	24,429	0.01%
clDNA	2,434	517,168	0.11%	2,491	487,816	0.12%
DNA/hAT	1,952	764,792	0.16%	1,818	675,712	0.17%
Total	189,829	131,302,944	27.76%	195,792	108,065,585	26.85%

### Microsatellite detection

Microsatellite (SSR) sequences have many advantages as molecular markers, due to their abundance, hypervariability, co-dominant nature, reliability, and ease of interpretation and several of groups have already identified SSR markers for *Musa*[37,60-63]. Type 1 SSRs (i.e. repeat sequences with a length greater than 20 bp), are considered to be hypervariable and the most efficient loci for use as molecular markers [64]. Analysis of the type I SSRs present in the A- and B-genomes demonstrates that the density of SSRs is slightly higher in the PKW B-genome at 1 per 323 bp, versus 1 per 387 bp in the ‘Pahang’ A-genome (Table 3), but that the proportions of microsatellite motifs were similar in both species. A comparison of the microsatelites identified by Hribova et al. [37] in ~100 Mb of repetitive sequences from *M. acuminata* cv. ‘Calcutta 4’ indicates a much higher proportion of trimeric and tetrameric motifs.

**Table 3:** **Overview of the microsatelites present in the ‘Pahang’ (Musa A) and ‘Pisang Klutuk Wulung’ (PKW, Musa B) genomes, and a comparison with the results of Hribova et al. obtained from the analysis of ~100 Mb of the repetitive region of *M. acuminata *cv ‘Calcutta 4’ genome**[37]

**Repeat size**	**Counts**					
	**Musa A**		**Musa B**		Musa A*	
	**‘Pahang’**	**%**	**PKW**	**%**	**‘Calcutta 4’**	**%**
2-mers	25,893	74	22,908	75	9,335	33
3-mers	5,245	15	4,571	15	8,988	32
4-mers	1,027	3	798	3	3,257	12
5-mers	505	1	390	1	1,923	7
6-mers	956	3	749	2	2,129	8
7-mers	266	1	220	1	797	3
8-mers	427	1	394	1	666	2
9-mers	192	1	225	1	555	2
10-mers	360	1	344	1	296	1
Total number of repeats	34,871		30,599		27,946	
Total number of repeat bases	2,095,593		1,955,541		1,003,716	
genome sized (bp)	472,960,417		341,431,243		98,538,911	
SSR bps/Kbp genome	4.44		5.73			
SSRs/Kbp genome	0.074		0.09			

* Results from Hribova et al. [37].

As shown in Table 3, dimeric repeat motifs are the most abundant type of class I SSR present in both *Musa* genomes, and TA the most common motif, representing 61.3 and 55.3% of all dimer repeats in the ‘Pahang’ and PKW genomes respectively (Table 4). The next most abundant dimer repeats were GA/TC at 35.5 and 41.3% and TG/CA at 3.1 and 3.35% respectively. A previous study of ‘Pahang’ BAC end sequences by Arango et al., also found AT/TA to be the most prevalent SSR representing ~26% of all SSR motifs [63], and characterization of the repeat component of ~30% of the *M. acuminata* cv. ‘Calcutta 4′ genome by low depth 454 sequencing also found TA and GA to be the most common dinucleotide repeats [37]. By comparison an extensive *in silico* study of EST databanks by Victoria et al. found AG/CT and GA/TC also to be the most common dimer motifs amongst vascular plant species [64]. The most abundant trimeric motifs in both *M. acuminata* and *M. balbisiana* genomes were ATT/AAT, ATA/TAT, GAA/TTC, and AAG/CTT, while Hribova et al. found GAA to be the most abundant trinucleotide motif in their study of the repetitive regions of the ‘Calcutta 4’ genome [37].

**Table 4:** **Overview of the dinucleotide microsatellite motifs present in the ‘Pahang’ (Musa A) and ‘Pisang Klutuk Wulung’ (PKW, Musa B) genomes, and a comparison with the results of Hribova et al. obtained from the analysis of ~100 Mb of the repetitive region of *M. acuminata *cv ‘Calcutta 4’ genome**[37]

**Motif**	**Musa A**		**Musa B**		**Musa A**	
	**‘Pahang’**		**PKW**		**‘Calcutta 4’**	
	**Count**	**%**	**Count**	**%**	**Count**	**%**
TA	15,885	61.35	12,676	55.34	5334	57.14
TC/GA	9206	35.56	9466	41.33	3228	34.58
TG/CA	801	3.1	766	3.35	0	0
AC/GT	0	0	0	0	765	8.2
CG	1	0.01	0	0	8	0.09
Total	25,893		22,908		9335	

Results from Hribova et al. [37].

### miRNA prediction

**Figure 2:**
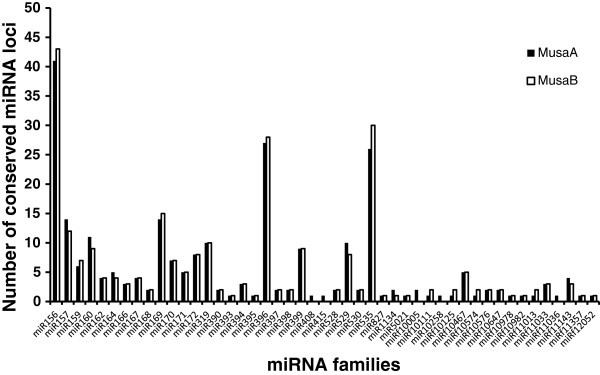
**Overview of numbers of conserved miRNA families present in the *Musa *A- and B-genomes.**

A non-redundant set of plant miRNAs, which included the 37 miRNA families (representing 234 precursors) previously reported for the ‘Pahang’ A-genome [14] was used to predict miRNA precursors and families within both *Musa* genomes (Figure 2). The results show a slightly larger number of predicted known miRNA precursors for the B-genome (270 miRNA precursors compared to 266 for the A genome), but the diversity of known miRNA families was lower, with 42 families predicted for the B-genome compared to 47 families for the A-genome. All of the known miRNA families detected in the B-genome were also found to be present in the A-genome. Overall, 10 additional miRNA families were found compared to those reported by D'Hont et al. [14]. The additional miRNA families detected were miR415, miR529, miR1134, miR5021, miRf10125, miRf10576, miRf11033, miRf11036, miRf11143 and miRf11357. Of these, only miR415 and miRf11036 were not detected in the B-genome. These new families may be due to additional entries added to the PMRD database since the analysis by D’Hont and colleagues, but could also be due to the new *M. acuminata* small RNA sequence data used in our query dataset. These were large (averaging >11 million Illumina sRNAseq “clean reads” per library) libraries, and derived from several different banana tissues (leaf, root, flower, fruit and somatic embryogenic cultures) - Additional file 6: Table S6.

As expected, the 42 miRNA families common to both genomes are shared with common ancestors of *Musa* i.e. embrophytes, angiosperms and poales. For example, the miR528 family which had previously been reported only for poales genomes, and recently shown by D’Hont et al. to be present in the A-genome [14] and is demonstrated here to also be present in the B-genome. Among the newly-predicted known miRNA families present in both *Musa* genomes, miR1134 has been reported as being abiotic stress - related and is found in the monocots *Triticum aestivum*[65] and *Festuca arundinacea*[66]; the miR5021 family has been reported to be pollen specific in *Arabidopsis*[67] whilst the families miRf10125, miRf10576, miRf11033, miRf11143 and miRf11357 are all of unknown function but were also computationally predicted from the *Arabidopsis*, poplar and rice genome sequences [68]. In addition to the known miRNA families, there were also 32 *Musa* miRNA precursors predicted, that belong to 28 novel miRNA families with no significant match to any previously reported mature miRNA sequence (Table 5). These include sequences that were unique to either *Musa* A- or B-genomes in addition to 4 families common to both genomes.

**Table 5:** Predicted novel miRNA in Musa A- and Musa B-genomes

**miRNA family**	**Locus A-genome**	**Locus B = genome**	**Mature_miRNA sequence 5′ – 3′**
mba-miR1		chr11: 8494985..8495047	AGAAACUUUUGUUGGAGAGGAAC
mac-miR2 mba-miR2	chr5:6134793..6134884	chr5: 5463327..5463417	CCGCAGGAGAGAUGAUGCCGCU
mba-miR3		chrUn_random:810706..810748	UACCGUACUGUACCGGCGUUU
mba-miR4		chr10: 20915746..20915798	CCUGAUUUGCUAAGUAGAUUU
mba-miR5		chr7: 16539311..16539413	UGGUUGAUGACGAUGUCGGCC
mac-miR6 mba-miR6	chr7:1377251..1377323	chr7: 1254647..1254718	UAGGAGAGAUGACACCGGCU
mac-miR7 mba-miR7	chr1:10560884..10561005	chr1: 9229340..9229458	AAACUAGUGCUAAGACCCAAUCUC
mba-miR8		chr1: 10667301..10667378	GGUGGUCUGGAUGAGGAUGCC
mac-miR9 mba-miR9	chr7:4569849..4569909	chr7: 4219274..4219333	UGGCUGAUGAUGAGUGAUCUU
mba-miR10		chr8: 20785187..20785267	CUUUGGCUUCUGGGUAGACGUA
mba-miR11		chr9: 8635028..8635089	UGUACGGAUAUGGUAGAGGGGCGU
mba-miR12		chr4: 17987962..17988016	AUCCCCGAGUGGGGUCGGUCGGAC
mba-miR13		chr8: 23338135..23338275	CUCGAGAUAUAUGAGUGUGGACA
mba-miR14		chrUn_random: 21488242..21488315	GGCACCUCGAUGUCGGCUC
mba-miR15		chr9: 25179371…25179434	GAGGAGGAGAAGAAAUGGAUCUG
mba-miR16		chr6: 3503608..3503675	GAAGAGGAAGGAGAAGUCG
mba-miR17		chr1: 17563902..17563986	CAGAAGUAGAAUACAUAAC
mba-miR18		chrUn_random: 135937842..135937988	UCCUUUUAGACCGUUGACGA
mac-miR19	chr4:22573796..22573893		UCCAGGAGAGAUGACACCAAC
mac-miR20	chr1:4998969..4999025		GGCGAUGAUGAUUGGUGAAUGU
mac-miR21	chrUn_random:15968301..15968390		GGAGAGAUGGCUGAGUGGACUAAA
mac-miR22	chrUn_random:23270434..23270480		CGAGGUGUAGCGCAGUCUGG
mac-miR23	chr8:31825102..31825180		UGGGAAGAAGACAAGGACAACAUG
mac-miR24	chr6:8249043..8249103		GAUCUCUGACCGAGCGGACUCC
mac-miR25	chr4:345377..345475		CAACGAUGAUGAGCCUACUAGACC
mac-miR26	chr11:15733314..15733359		AGAUGAGGUAAAGUAGUGCGA
mac-miR27	chr6:9168756..9168839		CAGCGACCUAAGGAUAACU
mac-miR28	chr7:28606179..28606234		GCGGAUGUGGCCAAGUGGU

### Genome distribution of miRNA precursors

**Figure 3:**
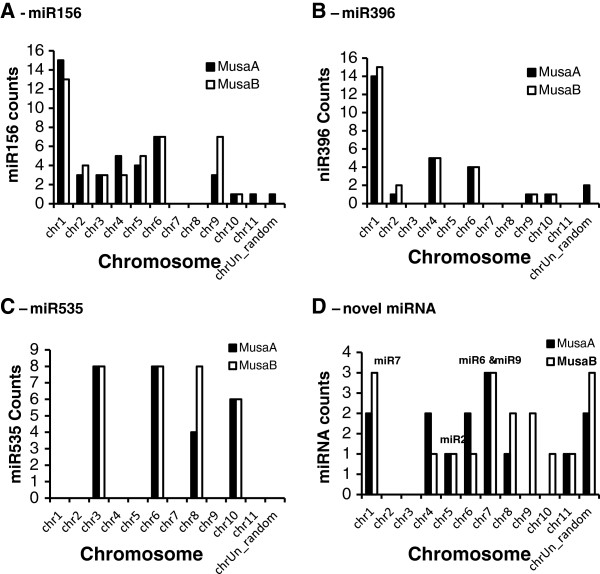
**Overview of the distribution of known and novel miRNA families over the Musa A- and B-genomes. A**. miR156, **B**. miR396, **C**. miR535, **D**. novel miRNA RNA families.

Although the overall B-genome size is smaller, it contains more repetitive elements (RE’s) as well as more known and novel miRNA (loci), but fewer conserved miRNA families than the A-genome. The three known miRNA families that were most highly represented in both *Musa* A- and B-genomes showed similar patterns of distribution across the chromosomes of both genomes, (Figure 2) suggesting synteny of A- and B-genomes. However, as the B-genome was assembled using the A-genome as reference, the gene order in this draft are preliminary and validation by FISH or similar methods will be needed to confirm this. As would be expected, the more recently evolved, novel miRNA families that are unique to either the *M. acuminata* or *M. balbisiana* genome are distributed fairly evenly across the genomes, with the exception of A-Chr2, B-Chr2, A-Chr3, B-Chr3, and A-Chr9 and A-Chr10, which lack any these sequences (Figure 3). The higher number of miRNA loci in the B-genome may be related to a higher number of transposable elements (transposons and retrotransposons) present as these are thought to have contributed to the generation of species-specific miRNA genes in plants [69]. The differences in retroelements and miRNA on homeologous chromosomes suggest therefore that some of these miRNAs have arisen after the whole genome duplication events, since chromosomes 9 and 10 (*Musa* block 2 of D’Hont et al. 2012) are among the regions thought not to have been involved in the paleopolyploidisation [14].

### Targets of novel B-genome miRNA

Using a fairly stringent cut-off expect value of 2.0, 18 predicted novel miRNA families were found in the B-genome. Of these, seven (mba-miR3, mba-miR5, mba-miR8, mba-miR12, mba-miR13, mba-miR15 and mba-miR18) were found to have targets within coding regions of the B-genome sequence. None of these predicted miRNA families were present in the A-genome [14] and thus are presumed to be B-genome specific in function and to have evolved after the divergence of the *M. balbisiana* and *M. acuminata* species ~4.6 Mya [54]. Predicted targets of these B-genome candidate miRNAs correlate with a range of functions across plant development and metabolism and notably, several are proposed to be involved in tolerance or responses to biotic and abiotic stress. These included casein kinase, a predicted target of mba-miR3 and previously reported to affect multiple developmental and stress response pathways in Arabidopsis [70]; dirigent, a predicted target of mba-miR15, which are a family of proteins associated with lignification, biotic and abiotic stress responses, also recently reported as responding to drought, salt and oxidative stresses in sugarcane [71] and mba-miR8 targets from the multidrug and toxic compound extrusion (MATE) family, which in plants are associated with tolerance to various xenobiotic compounds [72] and metals including zinc tolerance in Arabidopsis [73] and aluminium tolerance in sorghum [74]. An additional predicted target of mba-miR8, Sal-1 phosphatase, has been reported as a negative regulator of drought tolerance in Arabidopsis, acting via ABA-dependent and independent pathways [75].

Several of the B-genome specific miRNA predicted targets, are proteins with unknown function, and appear to be only present in the B-genome and thus may represent B-genome distinct functional networks. Given the wider stress and disease resistance reported for banana B genomes [23,76,77] further functional validation of these miRNA and target genes, and in particular those of unknown function, is of particular interest. A full list of predicted miRNA targets is provided in Additional file 7: Table S7.

### Annotating the PKW consensus B-genome

The results above demonstrate that the consensus genome derived from PKW gDNA mapping to the A-genome encompasses essentially all the genic B-genome regions. Analysis of the sequence variants and type 1 SSRs present in this consensus genome also produced results that show SSR and variant counts are comparable to those reported for the A-genome, and are in broad agreement with values obtained for other eukaryotic plant species. However, to be able to use the PKW genome sequences for molecular genetic research, gene annotation of the B-genome is obviously desirable. Therefore protein coding sequences in the B-genome were predicted by *ab initio* gene identification using the FGENESH software (http://linux1.softberry.com/all.htm). This resulted in the identification of 39,914 unique gene models. This number is higher than the 36,483 predicted from direct transfer of annotated regions of the A-genome to the B-genome and suggests that the gene count has been overestimated to the extent of around 109%. The higher predicted gene count is largely due to a nearly 2-fold higher number of gene models located within the concatenated contig set ‘B_chrUn_random’ , relative to the chrUn_random of the A-genome (Table 6). The set of PKW B-genome gene models were descriptively annotated online using the Blast2Go software [35,36]. Blast results against the NCBI non-redundant protein database show that 38,886 (97.4%) of the sequences had a positive hit, of which 30,541 had an e-value of 0. Following annotation steps, GO terms could be assigned to 37,367 (93.6%) sequences and 34,044 (85.3%) were annotated by interproscan. On the basis of the annotations assigned here, we can see that the B-genome contains 3,276 transposable elements (TE’s), of which 1,470 are located in the B-unChr_random sequence. If these TS’s are removed, the final functional B-genome gene count is actually 36,638, which is almost identical to the A-genome count of 36,542 of [14]. An overview and comparison of the A- and B-genomes and gene counts and annotations is provided in Table 6.

**Table 6:** **Comparison of the *Musa *A- and B-genome annotations**

**Information**	**A-chr1**	**A-chr2**	**A-chr3**	**A-chr4**	**A-chr5**	**A-chr6**	**A-chr7**	**A-chr8**	**A-chr9**	**A-chr10**	**A-chr11**	**A-chrUn_random**
Length	27,573,629	22,054,697	30,470,407	30,051,516	29,377,369	34,899,179	28,617,404	35,439,739	34,148,863	33,665,772	25,514,024	141,147,818
CDS	2,835	2,327	3,251	3,367	2,971	3,698	2,765	3,454	3,109	3,155	2,677	2,927
Gene	2,942	2,384	3,337	3,465	3,057	3,794	2,834	3,536	3,193	3,233	2,762	3,054
Polypeptide	2,835	2,327	3,251	3,367	2,971	3,698	2,765	3,454	3,109	3,155	2,677	2,927
mRNA	2,835	2,327	3,251	3,367	2,971	3,698	2,765	3,454	3,110	3,155	2,678	2,927
miRNA	31	16	21	39	22	16	14	14	22	12	13	15
**Information**	**B-chr1**	**B-chr2**	**B-chr3**	**B-chr4**	**B-chr5**	**B-chr6**	**B-chr7**	**B-chr8**	**B-chr9**	**B-chr10**	**B-chr11**	**B-chrUn_random**
Length	22,038,404	17,349,238	24,161,952	24,656,528	23,648,591	27,831,592	22,212,853	27,665,716	25,900,723	25,230,959	20,721,546	141,129,053
CDS	2,832	2,331	3,217	3,301	3,115	3,761	2,933	3,600	3,284	3,345	2,718	5,515
Gene	2,827	2,330	3,216	3,298	3,114	3,757	2,929	3,594	3,283	3,340	2,714	5,512
mRNA	2,827	2,330	3,216	3,298	3,114	3,757	2,929	3,594	3,283	3,340	2,714	5,511
miRNA	57	32	27	23	20	26	17	17	18	15	7	5

### RNA read and transcript mapping to the A- and B-genomes

To evaluate the usefulness and validity of the consensus PKW B-genome for expression studies, a total of 256 M paired-end, 100 bp RNA reads from two AAA, and three AAB cultivars, and one diploid *Austalimusa* Fe’i cultivar were mapped to the combined A- and B-genome sets. In addition we carried out *de novo* assembly of these reads to generate transcript sets for each cultivar, and these expressed gene transcripts were also mapped to the combined genomes set together with a ‘Grande Naine’ (AAA), Unigene set.

**Figure 4:**
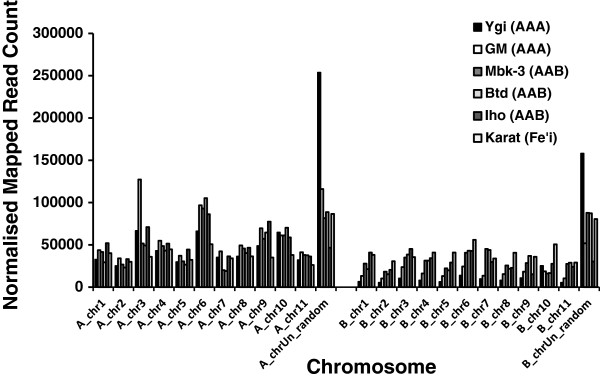
**Overview of distribution of mapped 100 bp RNA reads across individual chromosomes of the combined A- and B-genome sets.** RNA reads derived from fruit of each cultivar individually mapped. Ygi, AAA cultivar ‘Yangambi-km5’; GM, AAA cultivar ‘Gros Michel’; Mbk-3, AAB cultivar ‘Mbouroukou-3’; Btd, AAB cultivar ‘Batard’; Iho, AAB cultivar ‘Iholena lele’; Karat, diploid Fe’I cultivar ‘Karat’.

As expected, the proportion of mapped 100 bp reads generally decreased with the predicted genetic distance of the cultivar from the reference A-genome, with ‘Karat’ (Fe’i) having the lowest value (74.8%) and ‘Yangambi-km5’ (AAA) having the highest proportion at 90.7% (Additional file 8: Table S8). Although reads from the AAA cultivars should theoretically map only to the A-genome, we can see that 23 – 27% of the reads from the two triploid AAA cultivars preferentially map (have a higher homology) to regions of the B-genome, despite the apparent absence of a B-genome in these cultivars (Figure 4, Additional file 9: Table S9). This could reflect the different sub-group origins of these *M. acuminata* genomes. i.e. ‘Yangambi-km5’ , and ‘Gros Michel’ belong to the ‘Ibota’ and ‘Gros Michel’ *M. acuminata* subgroups respectively, whereas the reference ‘Pahang’ belongs to the *Malaccencis* subgroup. Also, differences between orthologous genes in these *M. acuminata* subgroup genomes could mean that only minor sequence divergence from ‘Pahang’ could lead to higher homology to the orthologous sequences present in the B-genome, particularly for highly-conserved ‘core’ genes. Alternatively the presence of foreign chromosomal fragments as a result of historical recombinations between the A- and B- genomes as demonstrated by Jeridi et al. [13], could result in the mapping of reads/transcripts to homologous regions of the homeologous B-chromosomes. By comparison, in the AAB plantains, we see that 36.4 – 40.7% of the reads from the three plantains preferentially map to the B-genome. This is in accordance with the presence of a single B-genome in these triploids, and confirms the utility of our PKW consensus B-genome sequence for this type of study. Finally, reads from the diploid Fe’i banana cultivar (*Musa, Australimusa,* 2n = 20), and a species which is probably most closely related to the wild species *M. maclayi*, *M. peekelii* and *M. lolodensis*[78], mapped nearly equally to both genomes (48.6 : 51.4, A B).

Interestingly, the normalized read coverage across the all chromosomes of both genomes was also found to differ between the cultivars. Of particular note are the differences between the two AAA hybrids (Figure 4, Additional file 9: Table S9), where 40.2% of all ‘Yangambi-km5’ mapped reads localized to the A- and B-chrUn_random’ sequences, compared to only 16.7% of the ‘Gros Michel’ reads. This suggests that there could be substantial differences between the AAA genome sequences of ‘Yangambi km-5’ and ‘Pahang’. However we also see that ‘B-chrUn_random’ is also the sequence with the highest count of TE sequences, so that these differences could simply represent differences in the abundance of these elements. Indeed comparing the genes with the highest expression levels in this chromosome in these 2 cultivars, shows that 250,822 reads specifically map to a single 472 bp intronic region of sequence (KMMuB_chrUn_random_G39317)_in ‘Yangambi km-5’ , while ‘only’ 60,891 map to this same sequence in ‘Gros Michel’. No such phenomenon was observed elsewhere however, where we see that a much higher proportion of ‘Gros Michel’ reads map to A_chr3 (Figure 4), and where for all cultivars a proportionally higher number of reads mapped to A_chr6, which is also the chromosome with the highest gene count (3,794, Table 6). However, even though the homeologous B_chr6 gene also has the highest gene count of the B-genome, more reads from the African plantains (‘Mbouroukou’ and ‘Batard’), mapped to B_chr7. These results indicate that there are different patterns of expression across the two chromosome sets (Figure 4), and suggest different and unique contributions of each genome to banana metabolism.

**Figure 5:**
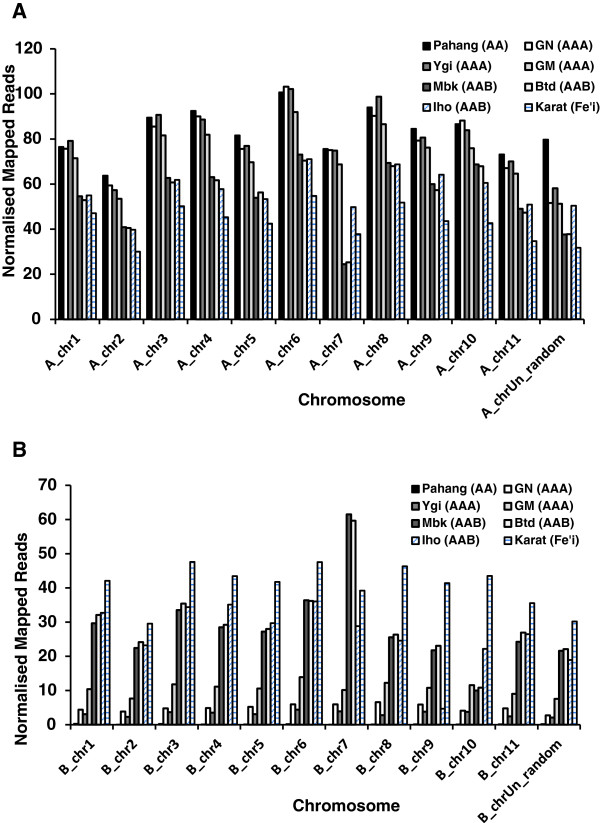
**Overview of distribution of mapped transcripts and ESTs across individual chromosomes of the combined A- and B-genome sets.** RNA transcripts were *de novo* assembled from 100bp RNA reads derived from fruit of each cultivar. ‘Pahang’, AA variety CDS reference set; Unigene’, EST set derived from AAA cultivar ‘Grande Naine’; Ygi, AAA cultivar ‘Yangambi-km5’; GM, AAA cultivar ‘Gros Michel’; Mbk-3, AAB cultivar ‘Mbouroukou-3’; Btd, AAB cultivar ‘Batard’; Iho, AAB cultivar ‘Iholena lele’; Karat, diploid Fe’i cultivar ‘Karat’.

The bias in gene coverage across the chromosomes of both genomes was examined further by looking at the mapping of the *de novo* assembled RNA contigs (transcripts) derived from the same RNA reads. Here the longer mean read length of the sequences improves the specificity of mapping and allows us to make a comparison with the results of the A-genome CDS and unigene mappings (Table 7, Figure 5).

**Table 7:** overview of RNA-transcript read coverage distribution per chromosome following mapping to the combined A- and B-genomes

**Sequence Name**	**Reference length/bp**	**Normalised mapped transcripts count**							
		**CDS**	**Unigenes**	**Ygi**	**GM**	**Mbk**	**Btd**	**Iho**	**Karat**
A_chr1	27,573,629	76.5	75.7	79.1	71.4	54.6	52.9	54.9	47.0
A_chr2	22,054,697	63.7	59.4	57.4	53.5	40.9	40.5	39.7	30.0
A_chr3	30,470,407	89.6	85.6	90.7	81.6	62.8	60.7	61.9	50.1
A_chr4	30,051,516	92.5	90.0	88.6	81.9	63.1	61.7	57.8	45.3
A_chr5	29,377,369	81.6	75.6	76.9	69.7	53.9	56.3	53.3	42.4
A_chr6	34,899,179	100.7	103.2	102.2	92.0	73.1	70.4	71.0	54.8
A_chr7	28,617,404	75.6	75.1	74.9	68.7	24.5	25.3	49.8	37.8
A_chr8	35,439,739	94.1	90.3	98.8	86.5	69.4	68.0	68.7	51.8
A_chr9	34,148,863	84.6	79.3	80.6	76.2	60.0	57.3	64.2	43.6
A_chr10	33,665,772	86.6	88.2	83.9	75.9	68.7	67.9	60.5	42.7
A_chr11	25,514,024	73.1	67.1	70.0	64.6	49.0	47.3	50.9	34.7
A_chrUn_random	141,147,818	79.7	51.6	58.1	51.3	37.7	37.8	50.4	31.7
B_chr1	22,038,404	0.3	4.4	3.1	10.4	29.6	32.1	32.7	42.1
B_chr2	17,349,238	0.1	3.8	2.3	7.6	22.4	24.1	23.2	29.6
B_chr3	24,161,952	0.3	4.8	3.7	11.8	33.5	35.4	34.4	47.6
B_chr4	24,656,528	0.1	4.9	3.5	11.1	28.5	29.2	35.1	43.4
B_chr5	23,648,591	0.1	5.2	3.0	10.6	27.2	28.0	29.7	41.7
B_chr6	27,831,592	0.2	5.9	4.4	13.9	36.4	36.2	36.1	47.6
B_chr7	22,212,853	0.1	5.9	3.9	10.2	61.5	59.6	28.8	39.2
B_chr8	27,665,716	0.1	6.6	2.8	12.2	25.6	26.3	24.6	46.3
B_chr9	25,900,723	0.1	5.9	3.8	10.8	21.8	23.0	4.7	41.4
B_chr10	25,230,959	0.1	4.1	3.8	11.6	10.0	10.8	22.2	43.5
B_chr11	20,721,546	0.1	4.8	2.5	9.0	24.3	26.9	26.5	35.6
B_chrUn_random	141,147,818	0.2	2.7	2.1	7.6	21.6	22.1	18.9	30.2
**% A**	**54.0**	**99.8**	**94.1**	**96.1**	**87.3**	**65.8**	**64.6**	**68.3**	**51.2**
**% B**	**46.0**	**0.2**	**5.9**	**3.9**	**12.7**	**34.2**	**35.4**	**31.7**	**48.8**

Read counts normalised per million total reads to allow direct comparison between cultivars. CDSm ‘Pahang’, AA variety CDS reference set; Unigenes’, EST set derived from AAA cultivar ‘Grande Naine’; Ygi, AAA cultivar ‘Yangambi-km5’; GM, AAA cultivar ‘Gros Michel’; Mbk-3, AAB cultivar ‘Mbouroukou-3’; Btd, AAB cultivar ‘Batard’; Iho, AAB cultivar ‘Iholena lele’; Karat, diploid Fe’i cultivar ‘Karat’.

Firstly, we see that 99.8% of the reference ‘Pahang’ CDS map to the A-genome, as expected. Similarly, 96.1% of the ‘Yangambi-km5’ (AAA) transcripts, and 94.1% of the ‘unigene’ sequences derived from “Grande Naine” (AAA, Cavendish sub-group) map to the B-genome (Table 6). Somewhat surprisingly though, only 87.3% of the ‘Gros Michel’ (AAA) transcripts map to the A-genome suggesting either that the ‘Ibota’ subgroup is more closely related to ‘Malacenssis’ subgroup to which ‘Pahang’ belongs than to the ‘Gros Michel’ subgroup, or that there is a larger proportion of *M. balbisiana*-like gene sequences in the ‘Gros Michel’ genome. Importantly the proportion of AAB transcripts mapping to the B-genome approaches the theoretically expected 33%, at 34.2 – 35.4% for the African plantains, but is slightly different than the 31.5% observed for Pacific plantain cultivar ‘Iholena lele’. Despite the fact that ‘Karat’ belongs to a completely different species (*Australimusa*), diploid ‘Karat’ transcripts map to both reference genomes equally well (51.2:48.8, A:B respectively), indicating that these resources could be useful to study gene expression even in *Australimusa* cultivars.

Looking at the mean normalized expressed transcript coverage per chromosome, it is clear that the high proportion of 100 bp reads mapping to the ‘chrUn_random’ sequences (Figure 4) is no longer evident. This suggests that the majority of the differences between cultivars involves non-expressed regions. For all cultivars the highest proportion of mapped reads are to be found on chromosomes A_chr6, and A_chr8, and the lowest number of mapped transcripts occurs on the two chrUn_random sequences. The African plantains are interesting in having a proportionally higher number of reads mapping to B_chr7 despite the low gene count of this chromosome. Some care has to be taken in the interpretation of differences in read distributions across the chromosomes as variations in physiological status (e.g. ripening) could lead to shifts in the mean mapped read depths per chromosomes. Nonetheless these results could provide indications as to those chromosomes in which there has been a higher degree of homeologous recombination, or which are preferentially expressed under certain conditions. The difference in the distribution of mapped ‘Iholena lele’ (Pacific plantain) transcripts compared to the African plantains, ‘Mbouroukou-3’ and ‘Batard’ supports their different classifications. i.e. even though all are hybrids between the *M. acuminata* subsp. *banksii* and *M. balbisiana* genomes [79], ‘Iholena lele’ is classified as a member of the Iholena and Maoli-Popoulu subgroup of plantains while the African plantains are considered to have arisen as a result of somaclonal mutations following a limited number of introductions to the African continent 2,300 – 2,750 years ago.

### Summary

The majority of modern cultivated banana cultivars are interspecific sterile triploids formed by hybridization between *M. acuminata* and *M. balbisiana* diploid progenitor species. Therefore the study of these hybrids ideally requires the presence of reference sequences for both genomes. In particular for the introgression of beneficial resistance traits associated with the *M. balbisiana* genome. Here, we generated a consensus B-genome sequence, which based on size estimates, represents ~78% of the expected B-genome length. This smaller size is presumably due to differences in the organisation of intergenic repetitive regions between the A- and B-genomes. This B-genome shows a high degree of sequence divergence from the A-genome as well as moderately high levels of heterozygosity. *Ab initio* gene prediction for the B-genome generates a total of 39,914 gene models, of which 3,540 are annotated as TE’s, so that the number of functional gene sequences is actually 36,638, which is nearly identical to the gene count of ‘Pahang’. We also identified several new B-genome specific miRNAs, some of which have predicted targets suggestive of novel stress related pathways that may have evolved separately in *M. Balbisiana*. To validate the usefulness of the consensus B-genome, mRNA-reads and *de novo* assembled transcripts from a series of genetically diverse interspecific triploid cultivars were mapped to the combined A- and B-genomes set. Results suggest significant genetic divergence between subgroups of AAA cultivars, and the presence of regions with high B-genome homology causing up to 12.7% of the transcripts to map preferentially to the B-genome. Comparison of the transcript mappings of the African and Plantain (AAB) varieties suggests structural diversity between these two groups, but importantly validate the quality of the B-genome, as ~33% of all reads map exclusively to these B-genome sequences. For all varieties there is evidence for differences in expression levels across homeologous chromosomes suggesting independent contributions of the two genomes to banana metabolism.

While our results demonstrate the usefulness of the consensus B-genome sequence for molecular genetic studies, it is important to relalise that the structure and organization is heavily dependent on the quality of the A-genome used as a reference. In this respect it should be remembered that only ~70% of the published A-genome sequence has actually been anchored to the 11 chromosomes. As a result, large scale structural differences in the B-genome are difficult to detect with our strategy, and intergenic regions of high diversity, and regions of the B-genome consisting of repeats will be difficult to assemble. In particular the position of the TEs, that represent around half of the *Musa* genome will be based on their position in the A-genome while in reality their position will in the vast majority of cases be different in the B genome.

## Conclusions

There is much interest in the exploitation of the *M. balbisiana* genome sequence for the introgression of beneficial traits such as biotic and abiotic stress resistance within *Musa* breeding programs. The PKW B-genome sequence therefore represents a valuable resource for the molecular genetic studies, not only in AAB, and ABB cultivars, but also for AAA dessert bananas. In addition we have shown that it can be used obtain useful information on gene expression levels in members of the more distantly-related *Australimusa* species.

### Data accession

The raw Illumina PKW gDNA sequence data generated was submitted to SRA with accession no. SRR956987. The assembled PKW consensus chromosome sequences, with annotations, as well as the CDS set and list of gDNA contigs are hosted at the Banana Genome Hub and publically available at http://banana-genome.cirad.fr/content/musa-balbisiana-pisang-klutuk--wulung.

## Competing interests

The authors declare that they have no competing interests.

## Authors’ contributions

MWD conceived and designed the PKW genome sequencing, carried out the read assembly, mapping and alignment, annotation, and variant calling and wrote the manuscript. WK helped draft the manuscript. LWS carried out the small RNA library preparation and initial analysis. RG carried out small RNA data analysis and participated in the sequence alignment and annotation. JAH and NK conceived the miRNA study design. RG and JAH helped to draft the manuscript. All authors read and approved the final manuscript.

## Supplementary Material

Additional file 1 CLC sequencing reads quality control report. Click here for file

Additional file 2: Table S2 Summary of *de novo* contig assembly of the unmapped, B-genome reads: In total 72% of the unmapped PKW 100 bp reads (2.611 Gbp) could be assembled into 63,245 contigs with an average length of 447 bp. The N50 or the scaffold size above which 50% of the total length of the sequence assembly can be found was 467 bp, with a maximum contig length of 17.328 Kb. 11,332 contigs had a length greater than 1 kb. The assembly parameters used were as follows; Word size: 25, Bubble size: 50, Minimum contig length = 200, Mismatch cost = 2, Insertion cost = 3, Deletion cost = 3, Length fraction = 0.5, Similarity fraction = 0.8. Mapping mode = Map reads back to contigs (slow). Click here for file

Additional file 3: Table S3 Summary of repeat annotation of *de novo* gDNA contigs assemblies derived from assembly of all unmapped PKW reads. Click here for file

Additional file 4: Table S4 Summary of *de novo* contig assembly of the all PKW B-genome 100 bp reads: In total 97.8% of the reads (26.535 Gbp) could be assembled into 180,175 contigs with an average length of 1.883 Kbp. The N50 or the scaffold size above which 50% of the total length of the sequence assembly can be found was 7.884 Kbp, with a maximum contig length of 152.268 Kb. 39,273 contigs had a length greater than 1 kb. The assembly parameters used were as follows; Word size: 25, Bubble size: 50, Minimum contig length = 200, Mismatch cost = 2, Insertion cost = 3, Deletion cost = 3, Length fraction = 0.5, Similarity fraction = 0.8. Mapping mode = Map reads back to contigs (slow). Click here for file

Additional file 5: Table S5 Summary of PKW genome variant analysis. Sequence variant analysis was carried out on both the RNA- and DNA-mappings using the ‘Probabilistic Variant Detection’ plugin in CLC Genomics Workbench, with settings specifying a minimum read coverage of 10 and a variant probability of >90%. The maximum expected number of variants was set as ‘2’ and variants detected using either all mapped reads (1) or ignoring non-uniquely mapped reads (2). Click here for file

Additional file 6: Table S6 Summary of small RNA libraries sequencing data. Click here for file

Additional file 7: Table S7 A full list of targets of predicted novel miRNA targets present in the PKW *M. balbisiana* genome. Click here for file

Additional file 8: Table S8 Overview of results following mapping of 100 bp paired RNA reads from 5 triploid hybrids and one Australimusa diploid cultivar against A- and B-genomes simultaneously. Ygi, AAA cultivar ‘Yangambi-km5’; GM, AAA cultivar ‘Gros Michel’; Mbk-3, AAB cultivar ‘Mbouroukou-3’; Btd, AAB cultivar ‘Batard’; Iho, AAB cultivar ‘Iholena lele’; Karat, diploid Fe’i cultivar ‘Karat’. Click here for file

Additional file 9: Table S9 Overview of the chromosomal distribution of 100 bp reads mapped to the combined A- and B- genomes simultaneously. Click here for file
