# Supplementary material for: “A draft Musa balbisiana genome sequence for molecular genetics in polyploid, inter- and intra-specific Musa hybrids”
Source: BMC Genomics. 2013 Oct 5;14:683. doi: 10.1186/1471-2164-14-683 (PMC3852598; doi:10.1186/1471-2164-14-683)
Supplement: Additional file 1 — CLC sequencing reads quality control report. [file 1471-2164-14-683-S1.pdf]

Sequencing QC Report
Based upon: 281,573,666 sequences in 1 data set
Generated by: markd
Creation date: Tue Sep 18 22:01:00 CEST 2012
Software: CLC Genomics Workbench 5.5.1

Table of contents

1. Summary 3
2. Per-sequence analysis 3
    2.1 Lengths distribution 3
    2.2 GC-content 4
    2.3 Ambiguous base-content 5
    2.4 Quality distribution 6
3. Per-base analysis 6
    3.1 Coverage 7
    3.2 Nucleotide contributions 8
    3.3 GC-content 9
    3.4 Ambiguous base-content 10
    3.5 Quality distribution 11
4. Over-representation analyses 11
    4.1 Enriched 5mers 12
    4.2 Sequence duplication levels 13
    4.3 Duplicated sequences 13

1. Summary

Creation date:	Tue Sep 18 22:01:00 CEST 2012
Generated by:	markd
Software:	CLC Genomics Workbench 5.5.1
Based upon:	1 data set
ITC1587_R1.MD_banana_BB.lane2.120817FCB (paired):	281,573,666 sequences in pairs

2. Per-sequence analysis

2.1 Lengths distribution

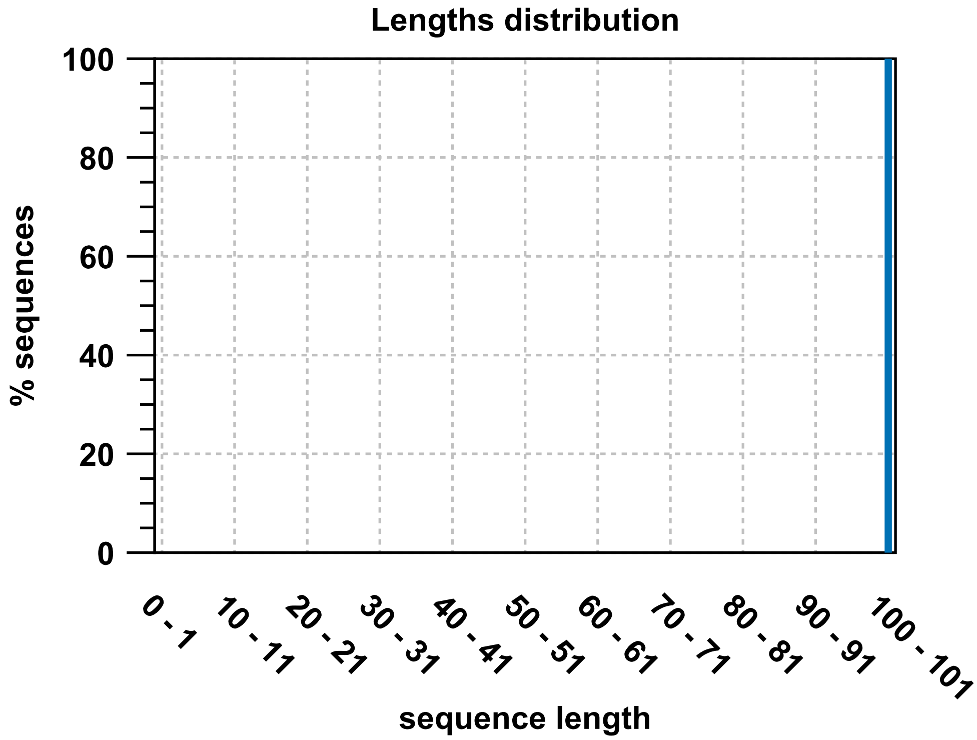

Distribution of sequence lengths. In cases of untrimmed Illumina or SOLiD reads it will ju st contain a single peak.
x: sequence length in base-pairs
y: number of sequences featuring a particular length normalized to the total number of seq uences

2.2 GC-content

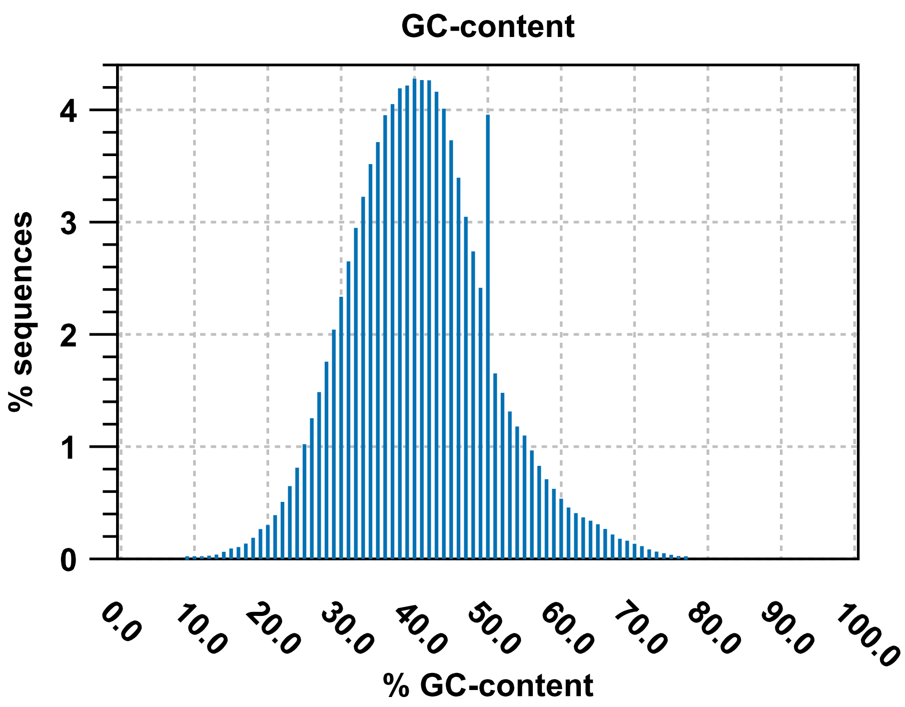

Distribution of GC-contents. The GC-content of a sequence is calculated as the number of G C-bases compared to all bases (including ambiguous bases).
x: relative GC-content of a sequence in percent
y: number of sequences featuring particular GC-percentages normalized to the total number of sequences

2.3 Ambiguous base-content

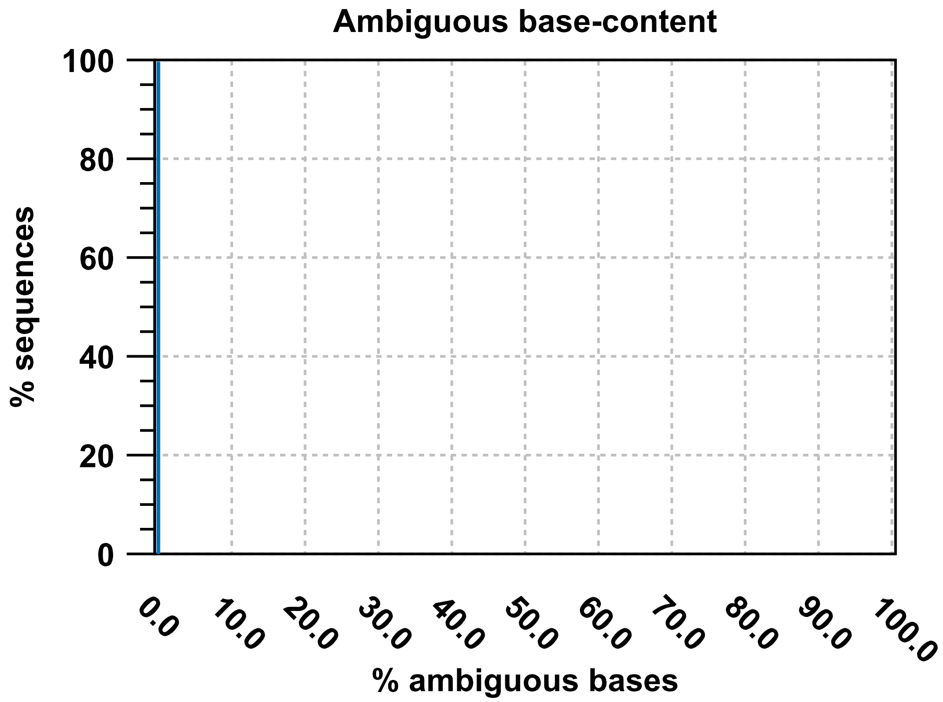

Distribution of N-contents. The N-content of a sequence is calculated as the number of amb iguous bases compared to all bases.
x: relative N-content of a sequence in percent
y: number of sequences featuring particular N-percentages normalized to the total number o f sequences

2.4 Quality distribution

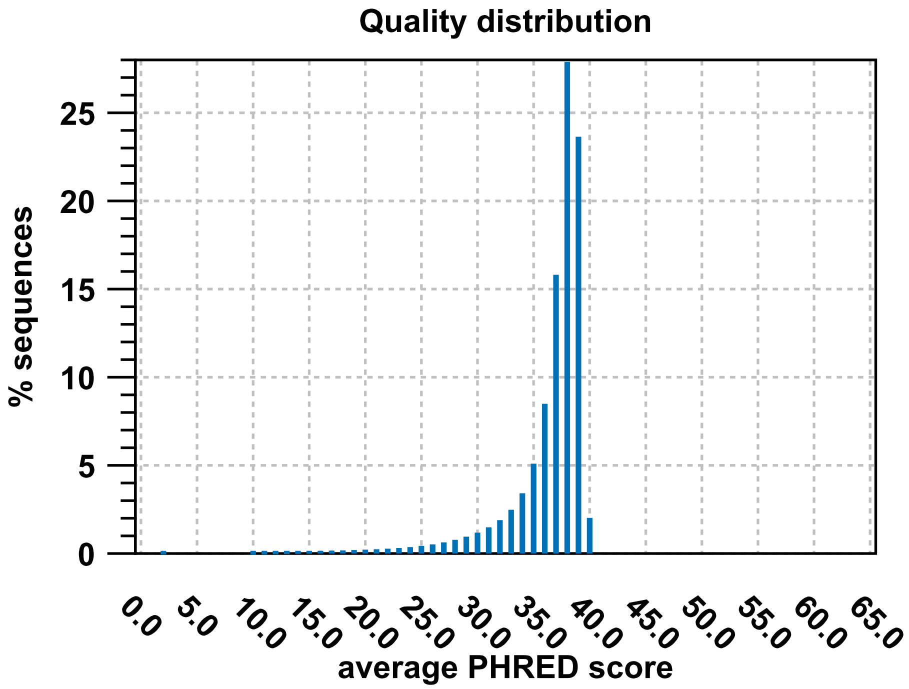

Distribution of average sequence qualitie scores. The quality of a sequence is calculated as the arithmetic mean of its base qualities.
x: PHRED-score
y: number of sequences observed at that qual. score normalized to the total number of sequ ences

3. Per-base analysis

3.1 Coverage

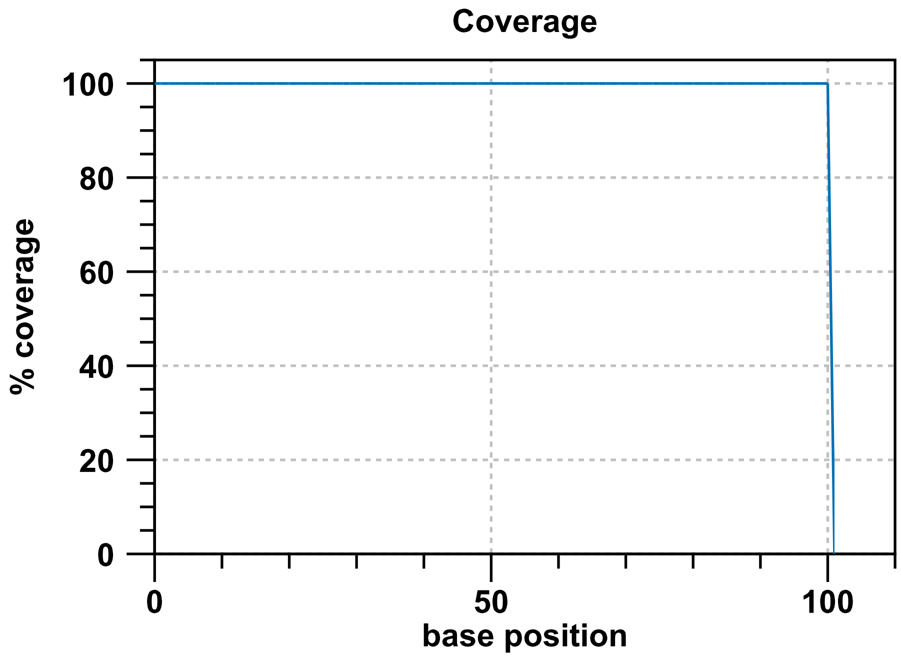


```
The number of sequences that support (cover) the individual base positions. In cases of un trimmed Illumina or
SOLiD reads it will just contain a rectangle.
x: base position
y: number of sequences covering individual base positions normalized to the total number o f sequences
```

3.2 Nucleotide contributions

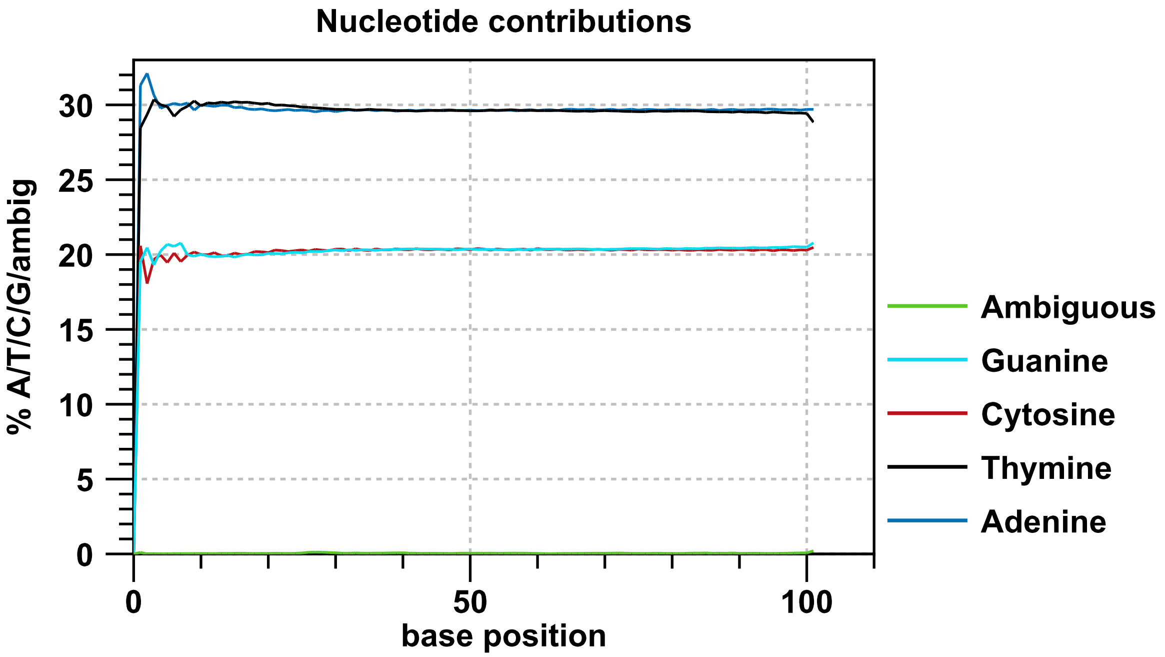


```
Coverages for the four DNA nucleotides and ambiguous bases.
x: base position
y: number of nucleotides observed per type normalized to the total number of nucleotides o bserved at that
position
```

3.3 GC-content

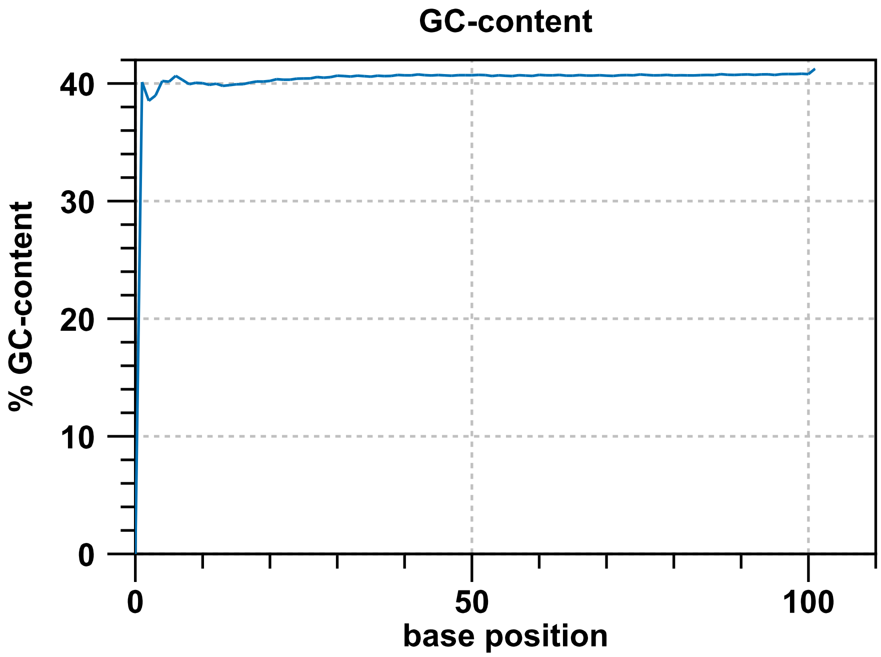

Combined coverage of G- and C-bases.
x: base position
y: number of G- and C-bases observed at current position normalized to the total number of bases observed at that position

3.4 Ambiguous base-content

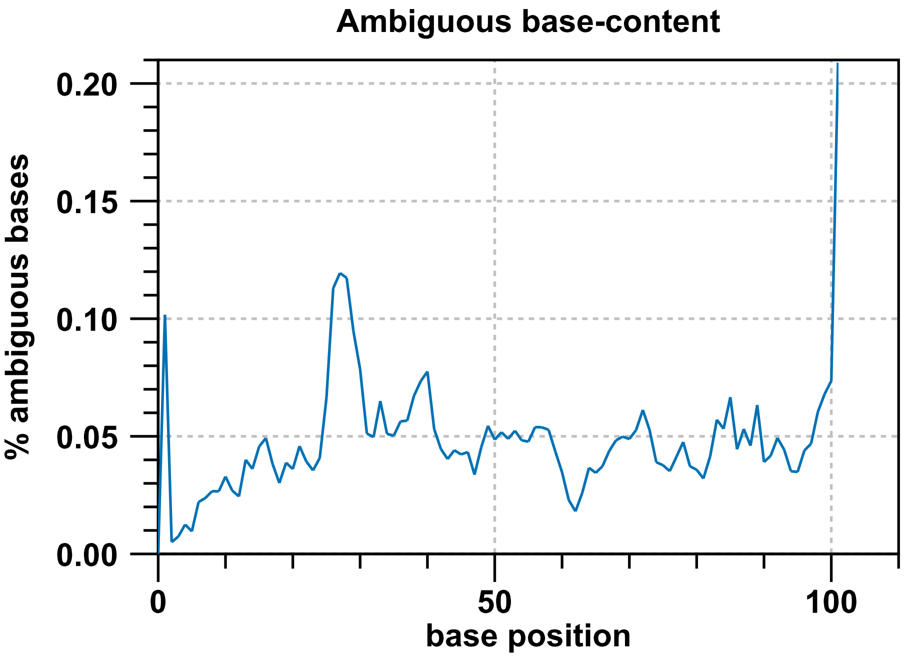

Combined coverage of ambiguous bases.
x: base position
y: number of ambiguous bases observed at current position normalized to the total number o f bases observed at that position

3.5 Quality distribution

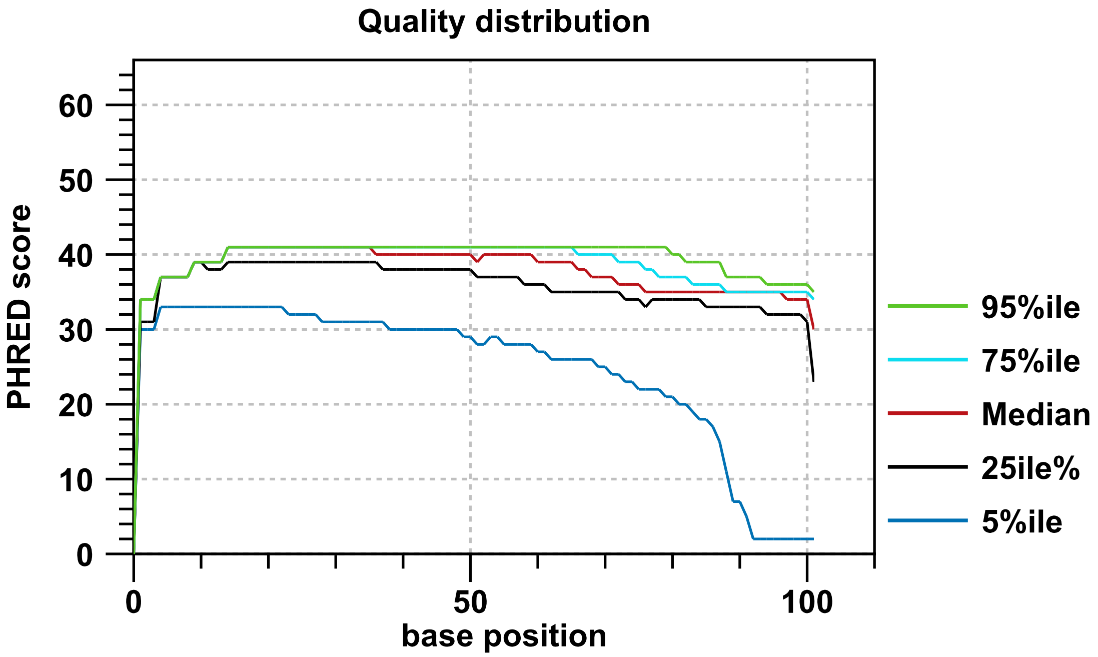


```
Base-quality distribution along the base positions.
x: base position
y: median & percentiles of quality scores observed at that base position
```

4. Over-representation analyses

4.1 Enriched 5mers

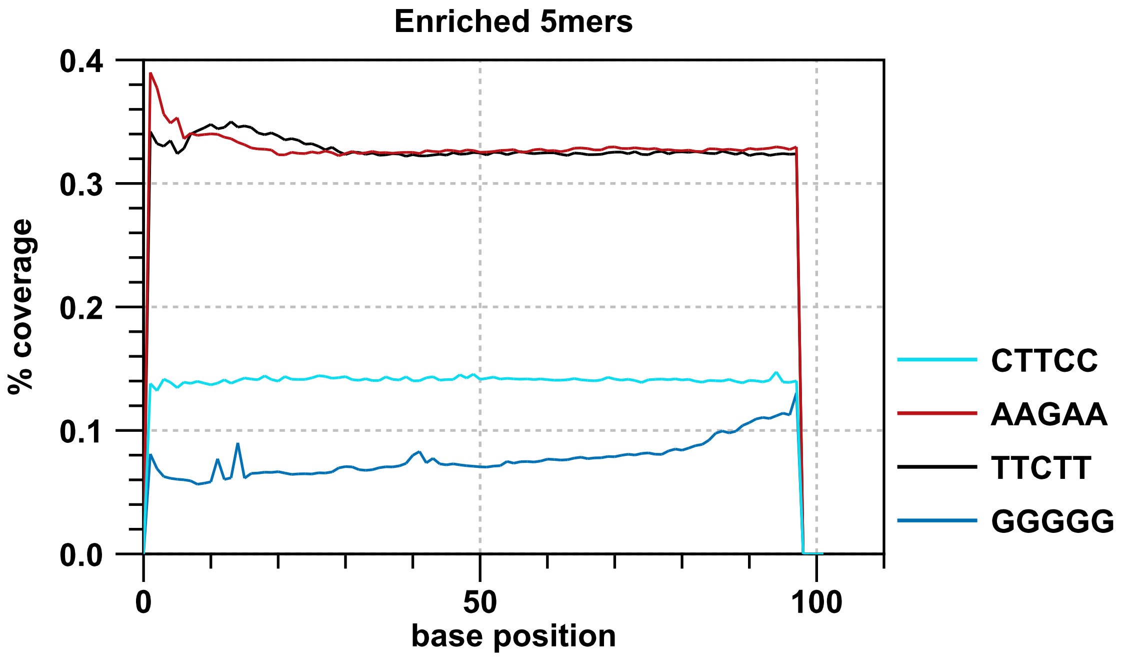

The five most-overrepresented 5mers. The over-representation of a 5mer is calculated as th e ratio of the observed and expected 5mer frequency. The expected frequency is calculated as product of the empirical nucleotide probabilities that make up the 5mer. (5mers that contain ambiguous bases are ignored)
x: base position
y: number of times a 5mer has been observed normalized to all 5mers observed at that posit ion

4.2 Sequence duplication levels

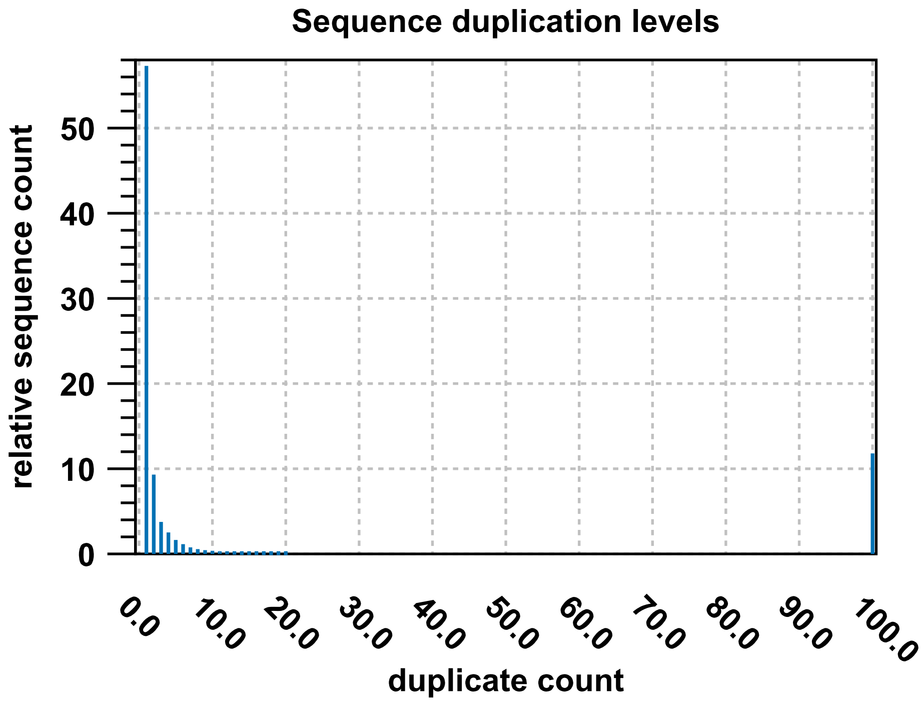

Duplication level distribution. Duplication levels are simply the count of how often a par ticular sequence has been found.
x: duplicate count
y: number of sequences that have been found that many times normalized to the number of un ique sequences

4.3 Duplicated sequences

A table of over-represented sequences is given in the supplementary report
