# Supplementary material for: “A draft Musa balbisiana genome sequence for molecular genetics in polyploid, inter- and intra-specific Musa hybrids”
Source: BMC Genomics. 2013 Oct 5;14:683. doi: 10.1186/1471-2164-14-683 (PMC3852598; doi:10.1186/1471-2164-14-683)
Supplement: Additional file 2: Table S2 — Summary of de novo contig assembly of the unmapped, B-genome reads: In total 72% of the unmapped PKW 100 bp reads (2.611 Gbp) could be assembled into 63,245 contigs with an average length of 447 bp. The N50 or the scaffold size above which 50% of the total length of the sequence assembly can be found was 467 bp, with a maximum contig length of 17.328 Kb. 11,332 contigs had a length greater than 1 kb. The assembly parameters used were as follows; Word size: 25, Bubble size: 50, Minimum contig length = 200, Mismatch cost = 2, Insertion cost = 3, Deletion cost = 3, Length fraction = 0.5, Similarity fraction = 0.8. Mapping mode = Map reads back to contigs (slow). [file 1471-2164-14-683-S2.doc]

**Supplementary table S2:** Summary of *de novo* contig assembly of the unmapped, B-genome reads:

#### In total 72% of the unmapped PKW 100bp reads (2.611 Gbp) could be assembled into 63,245 contigs with an average length of 447 bp. The N50 or the scaffold size above which 50% of the total length of the sequence assembly can be found was 467 bp, with a maximum contig length of 17.328 Kb. 11,332 contigs had a length greater than 1 kb. The assembly parameters used were as follows; Word size: 25, Bubble size: 50, Minimum contig length = 200, Mismatch cost = 2, Insertion cost = 3, Deletion cost = 3, Length fraction = 0.5, Similarity fraction = 0.8. Mapping mode = Map reads back to contigs (slow).

|  | | **Count/bp** | **Total bases** |
| --- | --- | --- | --- |
| Reads | | 36,834,390 | 3,630,032,943 |
| Matched | | 26,498,764 | 2,611,014,379 |
| % matched | | 72 |  |
| Not matched | | 10,335,626 | 1,019,018,564 |
| Contigs | N75 | 296 |  |
|  | N50 | 467 |  |
|  | N25 | 1,001 |  |
|  | Minimum | 135 |  |
|  | Maximum | 17,328 |  |
|  | Average | 447 |  |
| Total | | 63,245 | 28,285,614 |
