# Supplementary material for: “A draft Musa balbisiana genome sequence for molecular genetics in polyploid, inter- and intra-specific Musa hybrids”
Source: BMC Genomics. 2013 Oct 5;14:683. doi: 10.1186/1471-2164-14-683 (PMC3852598; doi:10.1186/1471-2164-14-683)
Supplement: Additional file 3: Table S3 — Summary of repeat annotation of de novo gDNA contigs assemblies derived from assembly of all unmapped PKW reads. [file 1471-2164-14-683-S3.doc]

**Supplementary table S3:** Summary of repeat annotation of *de novo* gDNA contigs assemblies derived from assembly of all unmapped PKW reads.

| **Class** | **PKW de novo gDNA contigs of unmapped reads** | | |
| --- | --- | --- | --- |
|  | **Count** | **Bp** | **%** |
| Ty1/Copia | 256 | 30,417 | 1.70% |
| Copia/Angela | 887 | 72,401 | 4.04% |
| Copia/SIRE1Maximus | 971 | 83,861 | 4.68% |
| Copia/Tnt1 | 234 | 23,793 | 1.33% |
| Ty3/Gypsy | 358 | 52,584 | 2.93% |
| Gypsy/CRM | 35 | 5,568 | 0.31% |
| Gypsy/Galadriel | 123 | 18,356 | 1.02% |
| Gypsy/Galadriel-lineage | 1 | 49 | 0.00% |
| Gypsy/Reina | 154 | 12,005 | 0.67% |
| Gypsy/Tekay | 268 | 37,010 | 2.06% |
| LINE | 144 | 13,694 | 0.76% |
| RE | 388 | 31,614 | 1.76% |
| Satellite/Type1 | 7 | 1,097 | 0.06% |
| Satellite/Type2 | 4 | 727 | 0.04% |
| clDNA | 813 | 139,458 | 7.78% |
| DNA/hAT | 51 | 6,156 | 0.34% |
| Total | 4,694 | 528,790 | 29.50% |
| Total Unmapped gDNA contigs annotated (63,245) | 3,565 | 1,792,490 | 5.60% |

Table S2 : Classification of repeats and the number of repeats in 15% PKW unmapped contigs.
