# Supplementary material for: “A draft Musa balbisiana genome sequence for molecular genetics in polyploid, inter- and intra-specific Musa hybrids”
Source: BMC Genomics. 2013 Oct 5;14:683. doi: 10.1186/1471-2164-14-683 (PMC3852598; doi:10.1186/1471-2164-14-683)
Supplement: Additional file 4: Table S4 — Summary of de novo contig assembly of the all PKW B-genome 100 bp reads: In total 97.8% of the reads (26.535 Gbp) could be assembled into 180,175 contigs with an average length of 1.883 Kbp. The N50 or the scaffold size above which 50% of the total length of the sequence assembly can be found was 7.884 Kbp, with a maximum contig length of 152.268 Kb. 39,273 contigs had a length greater than 1 kb. The assembly parameters used were as follows; Word size: 25, Bubble size: 50, Minimum contig length = 200, Mismatch cost = 2, Insertion cost = 3, Deletion cost = 3, Length fraction = 0.5, Similarity fraction = 0.8. Mapping mode = Map reads back to contigs (slow). [file 1471-2164-14-683-S4.doc]

**Supplementary table S4:** Summary of *de novo* contig assembly of the all PKW B-genome 100bp reads.

#### In total 97.8% of the reads (26.535 Gbp) could be assembled into 180,175 contigs with an average length of 1.883 Kbp. The N50 or the scaffold size above which 50% of the total length of the sequence assembly can be found was 7.884 Kbp, with a maximum contig length of 152.268 Kb. 39,273 contigs had a length greater than 1 kb. The assembly parameters used were as follows; Word size: 25, Bubble size: 50, Minimum contig length = 200, Mismatch cost = 2, Insertion cost = 3, Deletion cost = 3, Length fraction = 0.5, Similarity fraction = 0.8. Mapping mode = Map reads back to contigs (slow).

|  | | **Count/bp** | **Total bases** |
| --- | --- | --- | --- |
| Reads | | 281,252,266 | 27,361,169,977 |
| Matched | | 271,248,092 | 26,534,872,870 |
| % matched | | 96.4 |  |
| Not matched | | 10,004,174 | 826,297,107 |
| Contigs | N75 | 2,065 |  |
|  | N50 | 7,884 |  |
|  | N25 | 17,948 |  |
|  | Minimum | 64 |  |
|  | Maximum | 152,268 |  |
|  | Average | 1,883 |  |
| Total | | 180,175 | 339,338,356 |
