# Supplementary material for: “A draft Musa balbisiana genome sequence for molecular genetics in polyploid, inter- and intra-specific Musa hybrids”
Source: BMC Genomics. 2013 Oct 5;14:683. doi: 10.1186/1471-2164-14-683 (PMC3852598; doi:10.1186/1471-2164-14-683)
Supplement: Additional file 5: Table S5 — Summary of PKW genome variant analysis. Sequence variant analysis was carried out on both the RNA- and DNA-mappings using the ‘Probabilistic Variant Detection’ plugin in CLC Genomics Workbench, with settings specifying a minimum read coverage of 10 and a variant probability of >90%. The maximum expected number of variants was set as ‘2’ and variants detected using either all mapped reads (1) or ignoring non-uniquely mapped reads (2). [file 1471-2164-14-683-S5.doc]

**Supplementary table S5**: Summary of PKW genome variant analysis.

#### Sequence variant analysis was carried out on both the RNA- and DNA-mappings using the ‘Probabilistic Variant Detection’ plugin in CLC Genomics Workbench, with settings specifying a minimum read coverage of 10 and a variant probability of >90%. The maximum expected number of variants was set as ‘2’ and variants detected using either all mapped reads (1) or ignoring non-uniquely mapped reads (2).

| **Variants** | | **Counts-1 (all reads)** | **Counts-2 (ignore non-uniquely mapped reads)** |
| --- | --- | --- | --- |
| Total variants | | 21,643,087 | 20,657,389 |
| Total SNVs | | 19,820,141 | 18,868,899 |
|  | Homozygous SNVs | 8,891,551 | 8,738,760 |
|  | Heterozygous SNVs | 10,928,528 | 10,130,236 |
| Insertions | | 830,065 | 815,805 |
| Deletions | | 992,881 | 972,588 |
| Total variants in coding regions | | 4,929,038 | 4,880,516 |
|  | homozygous coding variants | 3,340,241 | 3,336,794 |
|  | heterozygous coding variants | 1,588,797 | 1,543,722 |
|  | Amino acid changes | 500,202 | 495,996 |
