# Supplementary material for: “A draft Musa balbisiana genome sequence for molecular genetics in polyploid, inter- and intra-specific Musa hybrids”
Source: BMC Genomics. 2013 Oct 5;14:683. doi: 10.1186/1471-2164-14-683 (PMC3852598; doi:10.1186/1471-2164-14-683)
Supplement: Additional file 6: Table S6 — Summary of small RNA libraries sequencing data. [file 1471-2164-14-683-S6.doc]

Supplementary table S6: Summary of small RNA libraries sequencing data.

| **sRNA libraries** | **Total read count** | **Unique read count** |
| --- | --- | --- |
| Root | 14,494,041 | 3,488,872 |
| Flower | 4,339,904 | 1,608,519 |
| Fruit | 4,926,076 | 2,526,944 |
| Leaves | 2,976,266 | 1,008,359 |
| Somatic Embryogenic cultures | 30,689,659 | 7,734,891 |
| Reads from all libraries | 57,425,946 | 16,367,585 |
| Non-redundant set  (size trimmed to 19-24nt) | 15,364,143 | 12,961,473 |
