# Supplementary material for: “A draft Musa balbisiana genome sequence for molecular genetics in polyploid, inter- and intra-specific Musa hybrids”
Source: BMC Genomics. 2013 Oct 5;14:683. doi: 10.1186/1471-2164-14-683 (PMC3852598; doi:10.1186/1471-2164-14-683)
Supplement: Additional file 7: Table S7 — A full list of targets of predicted novel miRNA targets present in the PKW M. balbisiana genome. [file 1471-2164-14-683-S7.doc]

Supplementary table S7 –A full list of targets of predicted novel *M. balbisiana* miRNA targets.

| **miRNA_Acc.** | **Target Gene Accession** | **Target Description** | **miRNA_Acc.** | **Target Gene Accession** | **Target Description** |
| --- | --- | --- | --- | --- | --- |
| Mba-miR3 | KMMuB_chr2_G03722 | ferredoxin-dependent glutamate synthase | Mba-miR15 | KMMuB_chr2_G03946 | leucine-rich repeat receptor-like protein kinase at2g19210-like |
|  | KMMuB_chr9_G25070 | casein kinase |  | KMMuB_chr9_G26004 | dirigent-like protein |
| Mba-miR5 | KMMuB_chr5_G12598 | alpha-amylase precursor |  | KMMuB_chrUn_random_G35259 | No annotation |
|  | KMMuB_chr7_G20691 | alpha-amylase precursor |  | KMMuB_chr6_G16374 | helicase domain-containing protein |
|  | KMMuB_chr5_G12404 | alpha-amylase precursor |  | KMMuB_chr10_G31031 | protein |
|  | KMMuB_chr5_G12400 | kinase interacting family protein |  | KMMuB_chr7_G20281 | hypothetical protein VITISV_041103 |
|  | KMMuB_chr10_G31545 | alpha-amylase precursor | Mba-miR18 | KMMuB_chr10_G28401 | hypothetical protein VITISV_043980 |
|  | KMMuB_chr8_G21814 | histone deacetylase 19-like |  | KMMuB_chr8_G23495 | retrotransposon-like protein |
|  | KMMuB_chrUn_random_G34767 | histone deacetylase 19-like |  | KMMuB_chrUn_random_G35706 | hypothetical protein VITISV_006955 |
| Mba-miR8 | KMMuB_chr1_G01417 | multidrug resistance |  | KMMuB_chr9_G26834 | retrotransposon ty1-copia subclass |
|  | KMMuB_chr10_G30154 | multidrug and toxin extrusion protein 1-like |  | KMMuB_chrUn_random_G34702 | frigida-like protein |
|  | KMMuB_chr3_G07121 | multidrug and toxin extrusion protein 2-like |  | KMMuB_chr9_G26967 | PREDICTED: uncharacterized protein LOC101218085 |
|  | KMMuB_chr4_G10476 | multidrug resistance |  | KMMuB_chr8_G23107 | PREDICTED: uncharacterized protein LOC101218085 |
|  | KMMuB_chr11_G31983 | multidrug resistance |  | KMMuB_chrUn_random_G35193 | No annotation |
|  | KMMuB_chr2_G03828 | sal1 phosphatase-like |  | KMMuB_chr7_G19362 | PREDICTED: uncharacterized protein LOC101218085 |
|  | KMMuB_chr6_G16462 | protein |  | KMMuB_chr7_G19340 | hypothetical protein VITISV_043746 |
|  | KMMuB_chr2_G03989 | multidrug and toxin extrusion protein 2-like |  | KMMuB_chr9_G26937 | retrotransposon-like protein |
|  | KMMuB_chr8_G22505 | multidrug resistance |  | KMMuB_chr10_G28978 | hypothetical protein VITISV_041588 |
|  | KMMuB_chr11_G32521 | protein |  | KMMuB_chrUn_random_G37729 | hypothetical protein VITISV_022540 |
|  | KMMuB_chr6_G17311 | sigma factor sigb regulation protein rsbq |  | KMMuB_chrUn_random_G36021 | hypothetical protein VITISV_030841 |
| Mba-miR12 | KMMuB_chr9_G26340 | retrotransposon ty3-gypsy subclass |  | KMMuB_chr8_G23163 | opie1 pol protein |
|  | KMMuB_chr1_G01861 | retrotransposon unclassified |  | KMMuB_chr3_G06388 | retrotransposon-like protein |
| Mba-miR13 | KMMuB_chr4_G11666 | transcription factor bhlh123-like |  | KMMuB_chr7_G20383 | phytoalexin-deficient 4-2 protein |
|  | KMMuB_chr7_G18824 | hydroxysteroid 11-beta-dehydrogenase 1-like protein |  | KMMuB_chrUn_random_G37096 | PREDICTED: uncharacterized protein LOC101218085 |
|  |  |  |  | KMMuB_chr4_G09928 | hypothetical protein VITISV_008903 |
|  |  |  |  | KMMuB_chr9_G27409 | hypothetical protein VITISV_012002 |
