# Supplementary material for: “A draft Musa balbisiana genome sequence for molecular genetics in polyploid, inter- and intra-specific Musa hybrids”
Source: BMC Genomics. 2013 Oct 5;14:683. doi: 10.1186/1471-2164-14-683 (PMC3852598; doi:10.1186/1471-2164-14-683)
Supplement: Additional file 8: Table S8 — Overview of results following mapping of 100 bp paired RNA reads from 5 triploid hybrids and one Australimusa diploid cultivar against A- and B-genomes simultaneously. Ygi, AAA cultivar ‘Yangambi-km5’; GM, AAA cultivar ‘Gros Michel’; Mbk-3, AAB cultivar ‘Mbouroukou-3’; Btd, AAB cultivar ‘Batard’; Iho, AAB cultivar ‘Iholena lele’; Karat, diploid Fe’i cultivar ‘Karat’. [file 1471-2164-14-683-S8.doc]

Supplementary table S8: Overview of results following mapping of 100bp paired RNA reads from 5 triploid hybrids and one Australimusa diploid cultivar against A- and B-genomes simultaneously. Ygi, AAA cultivar ‘Yangambi-km5’; GM, AAA cultivar ‘Gros Michel’; Mbk-3, AAB cultivar ‘Mbouroukou-3’; Btd, AAB cultivar ‘Batard’; Iho, AAB cultivar ‘Iholena lele’; Karat, diploid Fe’i cultivar ‘Karat’.

|  | **Counts** | | | | | |
| --- | --- | --- | --- | --- | --- | --- |
|  | **Ygi (AAA)** | **GM (AAA)** | **Mbk (AAB)** | **Btd (AAB)** | **Iho (AAB)** | **Karat** |
| Mapped reads | 39,388,133 | 47,568,053 | 24,306,101 | 45,231,059 | 51,671,817 | 30,180,343 |
| % mapped | 90.7 | 89.2 | 86.4 | 86.5 | 87.9 | 74.8 |
| Invalid mapped reads | 1,330,478 | 2,049,139 | 1,263,826 | 3,045,090 | 2,135,943 | 4,641,533 |
| Un-mapped reads | 2,695,736 | 3,697,978 | 2,564,981 | 4,019,484 | 5,001,526 | 5,486,084 |
| Reads in pairs | 16,481,324 | 20,943,062 | 14,615,332 | 18,053,474 | 23,251,546 | 11,201,982 |
| Broken paired reads | 22,786,111 | 26,524,073 | 9,598,244 | 27,050,730 | 28,293,686 | 18,851,072 |
| **Total reads** | **43,414,347** | **53,315,170** | **28,134,908** | **52,295,633** | **58,809,286** | **40,307,960** |
