# Supplementary material for: “A draft Musa balbisiana genome sequence for molecular genetics in polyploid, inter- and intra-specific Musa hybrids”
Source: BMC Genomics. 2013 Oct 5;14:683. doi: 10.1186/1471-2164-14-683 (PMC3852598; doi:10.1186/1471-2164-14-683)
Supplement: Additional file 9: Table S9 — Overview of the chromosomal distribution of 100 bp reads mapped to the combined A- and B- genomes simultaneously. [file 1471-2164-14-683-S9.doc]

Supplementary table S9:

#### Overview of read mapping to combined A- and B- genomes

| **Reference sequence** | **Reference length/bp** | **Counts** | | | | | |
| --- | --- | --- | --- | --- | --- | --- | --- |
|  |  | **Ygi** | **GM** | **Mbk** | **Btd** | **Iho** | **Karat** |
| A_chr1 | 27,573,629 | 33,218 | 43,791 | 42,105 | 29,676 | 51,289 | 39,586 |
| A_chr2 | 22,054,697 | 25,533 | 33,930 | 26,822 | 23,196 | 33,225 | 29,776 |
| A_chr3 | 30,470,407 | 69,141 | 129,620 | 52,625 | 51,007 | 71,914 | 36,704 |
| A_chr4 | 30,051,516 | 43,551 | 54,597 | 48,501 | 43,085 | 51,079 | 44,335 |
| A_chr5 | 29,377,369 | 30,201 | 36,980 | 30,618 | 26,466 | 44,460 | 32,205 |
| A_chr6 | 34,899,179 | 66,306 | 95,399 | 91,195 | 102,432 | 84,937 | 50,535 |
| A_chr7 | 28,617,404 | 35,656 | 42,124 | 20,189 | 19,305 | 36,244 | 33,762 |
| A_chr8 | 35,439,739 | 36,873 | 49,014 | 45,743 | 40,380 | 46,621 | 36,488 |
| A_chr9 | 34,148,863 | 49,532 | 69,330 | 57,577 | 65,709 | 76,659 | 35,301 |
| A_chr10 | 33,665,772 | 64,758 | 60,862 | 60,841 | 71,171 | 58,225 | 37,743 |
| A_chr11 | 25,514,024 | 32,789 | 41,094 | 38,247 | 37,564 | 36,094 | 26,558 |
| A_chrUn_random | 141,147,818 | 246,335 | 111,943 | 78,996 | 85,374 | 45,583 | 82,988 |
| **Total counts A** | **472,960,417** | **733,892** | **768,683** | **593,458** | **595,366** | **636,330** | **485,981** |
| B_chr1 | 22,038,404 | 6,692 | 13,707 | 28,421 | 21,630 | 41,074 | 38,756 |
| B_chr2 | 17,349,238 | 5,673 | 10,366 | 18,451 | 15,483 | 20,611 | 30,549 |
| B_chr3 | 24,161,952 | 10,638 | 23,807 | 35,253 | 39,038 | 45,193 | 35,122 |
| B_chr4 | 24,656,528 | 8,426 | 16,527 | 31,683 | 31,368 | 34,347 | 40,653 |
| B_chr5 | 23,648,591 | 6,405 | 13,294 | 22,343 | 19,877 | 29,153 | 39,977 |
| B_chr6 | 27,831,592 | 14,479 | 25,547 | 41,105 | 43,153 | 43,341 | 56,708 |
| B_chr7 | 22,212,853 | 9,977 | 13,700 | 45,482 | 43,992 | 29,802 | 33,865 |
| B_chr8 | 27,665,716 | 8,286 | 15,816 | 25,849 | 21,842 | 22,887 | 41,297 |
| B_chr9 | 25,900,723 | 11,136 | 18,519 | 28,904 | 37,947 | 15,447 | 36,274 |
| B_chr10 | 25,230,959 | 24,922 | 18,351 | 16,216 | 16,568 | 27,359 | 48,659 |
| B_chr11 | 20,721,546 | 5,556 | 10,696 | 27,590 | 28,746 | 24,208 | 29,697 |
| B_chrUn_random | 141,147,818 | 153,921 | 50,986 | 85,244 | 84,990 | 30,248 | 82,463 |
| **total counts B** | **341,431,243** | **266,111** | **231,316** | **406,542** | **404,634** | **363,670** | **514,019** |
| **% reads mapped A** | **58.1** | **73.4** | **76.9** | **59.3** | **59.5** | **63.6** | **48.6** |
| **% reads mapped B** | **41.9** | **26.6** | **23.1** | **40.7** | **40.5** | **36.4** | **51.4** |
